# Ventx Family and Its Functional Similarities with Nanog: Involvement in Embryonic Development and Cancer Progression

**DOI:** 10.3390/ijms23052741

**Published:** 2022-03-01

**Authors:** Shiv Kumar, Vijay Kumar, Wenchang Li, Jaebong Kim

**Affiliations:** 1 School of Psychology and Neuroscience, University of St Andrews, St. Mary’s Quad, South Street, St. Andrews KY16 9JP, UK; wl21@st-andrews.ac.uk; 2 Department of Biochemistry, Institute of Cell Differentiation and Aging, College of Medicine, Hallym University, Chuncheon 24252, Gangwon-Do, Korea; vijay10187@gmail.com

**Keywords:** embryonic development, homeobox Nanog, genome evolution, tumorigenesis, Ventx family, *Xenopus*, zebrafish

## Abstract

The Ventx family is one of the subfamilies of the ANTP (*antennapedia*) superfamily and belongs to the NK-like (NKL) subclass. Ventx is a homeobox transcription factor and has a DNA-interacting domain that is evolutionarily conserved throughout vertebrates. It has been extensively studied in *Xenopus*, zebrafish, and humans. The Ventx family contains transcriptional repressors widely involved in embryonic development and tumorigenesis in vertebrates. Several studies have documented that the Ventx family inhibited dorsal mesodermal formation, neural induction, and head formation in *Xenopus* and zebrafish. Moreover, Ventx2.2 showed functional similarities to Nanog and Barx1, leading to pluripotency and neural-crest migration in vertebrates. Among them, Ventx protein is an orthologue of the Ventx family in humans. Studies have demonstrated that human Ventx was strongly associated with myeloid-cell differentiation and acute myeloid leukemia. The therapeutic potential of Ventx family inhibition in combating cancer progression in humans is discussed. Additionally, we briefly discuss genome evolution, gene duplication, pseudo-allotetraploidy, and the homeobox family in *Xenopus*.

## 1. Introduction

**Figure 1:**
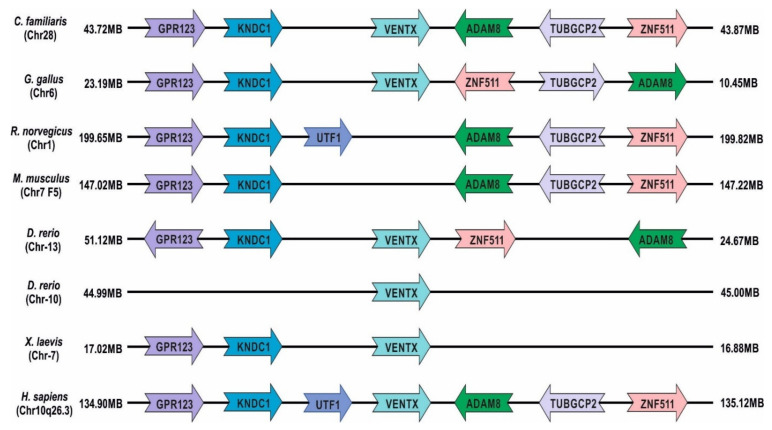
Chromosomal localization of Ventx gene synteny in humans and other animal species relative to adjacent genes (color-coded).

*Xenopus laevis* is an established animal model for studying vertebrate development, neurobiology, immunology, cell biology, and toxicology [1,2]. *Xenopus* is a pseudo-tetraploidy organism [3,4]. Its genome tetraploidy arose from the merging of two progenitor ancestral frog genomes millions of years ago [3,4,5,6]. During genome evolution, the *Xenopus laevis* genome was subdivided into a longer subgenome (L-subgenome) and a shorter subgenome (S-subgenome). There is some gene duplication. However, most of the functional genes are located in the L-subgenome, while the majority of non-functional genes or pseudogenes are in the S-subgenome, formed by either the deletion of exons or frameshifts by single-nucleotide insertion/deletion [4,5,6,7]. The presence of non-functional genes or pseudogenes leads to doses compensation. Many families of transcriptional factors have evolved in vertebrates to regulate a wide network of downstream genes in a temporospatial-dependent manner to orchestrate orderly embryonic development and organogenesis [8,9,10]. Among those families, the homeobox domain-containing families, including the Ventx family, play crucial roles during embryonic development and are widely involved in tumorigenesis in vertebrates [9,11]. The Ventx family is a transcription-repressor family belonging to the NKL subclass. The NKL subclass is one of the subclasses of ANTP (the *antennapedia* gene, one of the Hox genes identified within the ANT-C homeotic complex of Drosophila) superfamily [8,9,11]. The Ventx family has been extensively studied in *Xenopus*, zebrafish, and humans. BMP4/Smad1 and FGF/Xbra signaling initiates the expression of the Ventx family members in *Xenopus* [12,13,14,15]. Recent studies have shown that BMP4/Smad1 and FGF/Xbra controlled the Ventx family expression in both *Xenopus* [13] and humans [16,17,18]. BMP4/Smad1 is involved in a signaling path leading to the positive regulation of the expression of Vega family genes (a homolog of Ventx family in *Xenopus*) in zebrafish [19,20,21]. Smad1 and *Xenopus* brachyury (Xbra) physically interact within the 5′-upstream regions of *ventx1.1* and *ventx2.1*, either in combination or separately, to augment *ventx1.1* and *ventx2.1* transcription in *Xenopus* [13,14,16,22,23,24,25,26]. In addition, Ventx2.2 is an ortholog of Nanog in *Xenopus* and exhibited a sequential and functional similarity to Nanog and Barx1 [27,28]. Although studies have revealed that Ventx synteny has been evolutionarily conserved in cats (Chr28), humans (Chr10, q26.3 loci), *Gallus* (Chr6), zebrafish (Chr13 and Chr10), and *Xenopus* (Chr7), it is absent in rodents, including rats and mice (Figure 1) [13,29]. Ventx family genes are clustered at chromosome 7 in *Xenopus*, though it has been identified as non-clustered in zebrafish (Chr13 and Chr10). In humans, seven pseudogenes of the Ventx family have been discovered, dispersed throughout different chromosomal locations, including VENTXP1 (Xp21.3), VENTXP2 (13q31.1), VENTXP3 (12q21.1), VENTXP4 (3p24.2), VENTXP5 (8p12), VENTXP6 (8q21.11), and VENTXP7 (3p24.3) [11]. The Ventx family proteins play various roles during axial patterning, germ cell specification, and cell differentiation during the embryonic development of vertebrates [30,31,32,33,34,35,36,37,38]. Additionally, the Ventx family members trigger ventral mesoderm, ectoderm, and ventral-blood-island (VBI) formations [22,30,35,39,40,41,42]. In contrast, increased levels of the Ventx family directly suppresses the dorso-anterior development, inhibiting head formation and primary neurogenesis in *Xenopus* and zebrafish [15,30,33,34,38,43,44,45]. In *Xenopus*, the ectopic expression of Ventx family members attenuated the expression of organizer-specific genes, including *goosecoid* (*gsc*), *chordin*, *noggin*, and *foxd4l1.1b* (an early neural marker); and the same occurred for *zic3* (a neuroectodermal marker) in *Xenopus* embryos at St.11 [13,30,33,34,35,45,46,47]. This attenuation caused the inhibition of dorsal mesoderm formation and neuroectodermal induction. The suppressed expression of organizer-specific genes resulted in a headless phenotype. Studies have shown that Ventx2.2/Xom acts as a Nanog-like pluripotency marker in *Xenopus* [48,49]. Overexpressed Ventx2.2 stabilizes the cells’ pluripotency and inhibits multiple-cell-lineage commitment during the embryonic development of *X. laevis*.

Similarly to *Xenopus*, BMP4/Smad1 controls the expression of the Vega family members [34,38,45]. The ectopic expression of Vega family members diminishes the dorsal and organizer-specific markers, leading to embryonic ventralization. Studies have found elevated levels of Ventx in acute-myeloid-leukemia (AML) cells, macrophages, and dendritic cells to promote their differentiation and leukemogenesis in humans and animal models [18,32,50,51]. Studies have shown that p53 transactivates the expression of p21 (p21^Cip1/Waf1^, an endogenous cell-cycle inhibitor) to inhibit the activity of cyclin–CDK complexes, including cyclin–Cdk1, cyclin–Cdk2, and cyclin–Cdk4/6 during G1 and S phases [52,53]. During DNA damage response, p53–p21 leads to cell senescence by inhibiting the CDK2/4–cyclinE/D1 complex, which arrests cell cycles. In contrast, Wu et al. (2011) revealed that Ventx directly transactivated the tumor suppression pathways in a p53–p21 and p16ink4a-Rb (a molecular link protein between cell senescence and tumor suppression)-dependent manner, leading to cellular senescence [54]. These studies suggested that the Ventx family plays multifaceted roles in hematopoietic cell differentiation and AML progression, tumor suppression, and apoptotic-pathway activation. We review these roles of the Ventx family in *Xenopus*, zebrafish, and humans and discuss the therapeutic potential of utilizing the Ventx family crosstalk pathways to inhibit cancer progression in humans. Additionally, we discuss the pseudo-tetraploidy, genome evolution, and gene duplication in *Xenopus*, *Xenopus*’s classes of the homeobox superfamily, and functional similarities of Ventx2.2 to Nanog and Barx1.

### 1.1. Xenopus laevis: Genome Evolution, Pseudo-Tetraploidy, and Gene Duplication

Polyploidization has been found in many animal species from annelids to insects, including some fish and amphibians [55]. However, polyploid animal species are relatively rare, as compared to those found in plants, and the possible barriers to generating polyploidization include the presence of sex chromosomes, the prevalence of cross-fertilization, and the histological complexity in evolutionally advanced animals. Despite these barriers, hermaphroditic polypoid species of amphibians continue to be discovered. Among vertebrates, the only diploid and polyploid hermaphroditic species are amphibians. (For reviews, see Schmid M. et al., 2015, and Becak et al., 2014 [55,56]). Two *Xenopus* species (i.e., *X. laevis* and *X. tropicalis*) have been used extensively for developmental, toxicological, immunological, genetic, and neurobiological studies [1,2]. *Xenopus laevis* is a polyploid species and an ideal model for studying the genome duplication. *Xenopus laevis* contains 36 chromosomes, nearly twice as many as in its close relative diploid *X. tropicalis* with 20 chromosomes [7]. Studies have reported that the allotetraploidy of *X*. *laevis* began by genome partitioning into two homoeologous S-subgenomes that then evolved separately and asymmetrically around 34 million years ago [6,57]. Around 17–18 million years ago, the genomes of these two diploid progenitor species combined to form an allotetraploid species (*X. laevis*) [5,6]. These two subgenomes are distinct but share the same nucleus (separate recombination identities). After the genomic merge, more than 56% of the genes were retained in two homoeologous copies on L and S chromosomes [6]. The L-subgenome retains most of the functional genes and more consistently resembles its progenitor’s condition, whereas the S-subgenome contains the majority of non-functional or pseudogenes due to deletions, insertions, rearrangements, and reduced expression. Therefore, *X. laevis* is considered a pseudo-tetraploid species. This asymmetry between the L and S-subgenomes could possibly have resulted from the intrinsic differences between their ancestral diploid progenitors. Additionally, the activation of transposable-elements-based remodeling of the S-subgenome might have been responsible for the L–S genome divergence itself. The L-subgenome of *X. laevis* is more closely related to the human genome than the S-subgenome of *X. tropicalis* [6]. A study reported that a single gene of *X. tropicalis* corresponded to precisely two co-orthologs in *X. laevis*, yet showed similar functionality in *X. laevis* [4]. The same study tested more than 2200 cases of gene duplication to show that the *X. laevis* genome possessed two co-orthologs of a gene in *X. tropicalis*. In contrast, single copies of *X. laevis* genes did not show similar outcomes to the ortholog of *X. tropicalis*. Duplicated genes can differ in expression levels and patterns, but not in molecular and biological functions. The duplication of gene sets is retained through a process of subfunctionalization (dosage compensation) and/or the relaxation of constraints on both copies of ancestral genes [4]. *X. laevis* thus provides a model for testing theories of whole genome duplication and significance, as its tetraploidization is neither too recent for studying the functions of a duplicated locus nor too ancient for measuring the impacts of single-nucleotide polymorphisms (SNPs) [4]. In addition, a recent genome-wide comparative study of aquaporins (AQPs) in allotetraploid *X. laevis* and diploid *X. tropicalis* revealed that the number of AQPs in *X. laevis* was nearly double (32) that of *X. tropicalis* (19) [3]. Meanwhile, a synteny-based analysis showed that the L-subgenome contained 17 AQPs, while the shorter S-subgenome had 15 AQPs. Additionally, six members of AQPs have been lost through evolution (i.e., two from the L-subgenome and four from the S-subgenome). These lost members are pseudogenized via either exon deletion or frameshift insertion and deletion. The merging of two ancestral genomes in the allotetraploid *Xenopus laevis* and the evolution of non-functional gene-duplication has indicated that *Xenopus laevis* is a pseudo-allotetraploid organism [2,3,5,6,7,55,57]. Consequently, the allotetraploidy of *Xenopus laevis* genome has become an important model in which to study polyploidy and aneuploidy formation. Tetraploidy and aneuploidy have been reported as having associations with various kinds of diseases in humans, such as cancers, Parkinson’s, aging disorders, Alzheimer’s, and neuromuscular developmental disorders [58,59,60,61,62,63,64,65,66].

### 1.2. Homeobox Domain (HD)

**Figure 2:**
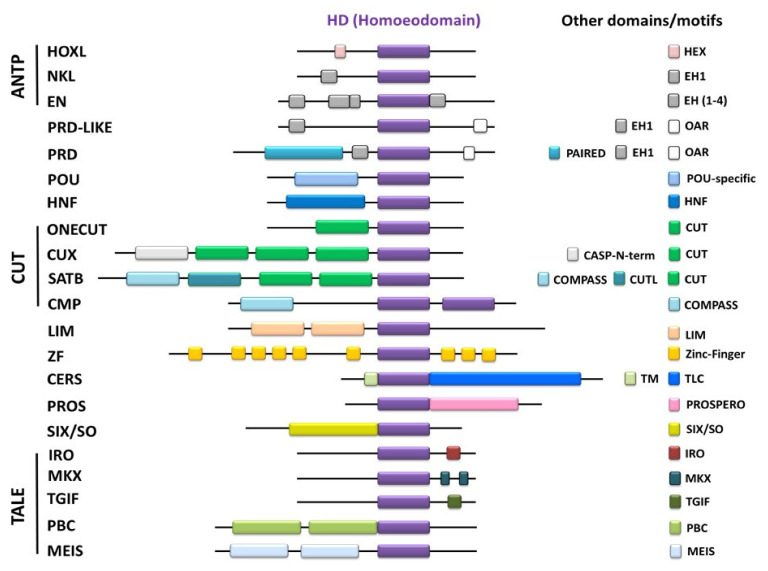
Schematic representation of conserved domains and motifs associated with different classes of HD proteins in animals. This figure was adapted from Burglin et al. (2016) [9].

Homeobox domain is a well-conserved 60-aa-long DNA-binding domain (except the TALE superclass, which contains 63 aa instead of 60 aa), identified first in *Drosophila* [10,67]. In the last two decades, our understanding of the homeobox domain has expanded, and a large number of homeobox proteins in plants and animals have been discovered (see Burglin and Affolter 2016 for review) [9]. There are 16 known major classes in animals, including ANTP (HOXL and NKL), PRD, PRD-like, POU, HNF, CUT (subclasses ONECUT, CUX, SATB, CMP), LIM, ZF, CERS, PROS, and SIX/SO, along with the TALE superclass (subclasses IRO, MKX, TGIF, PBC, MEIS) (Figure 2). These homeobox proteins play diverse biological roles during development, such as in cell proliferation, pluripotency, and differentiation in a temporospatial and context-dependent manner [9]. The HD proteins are either transcription activators or repressors that directly interact with the 5′-upstream regions of genes to modulate gene transcription. ANTP inherited its name from the *antennapedia* (ANTP) gene, one of the Hox genes identified within the ANT-C homeotic complex of Drosophila [11]. The ANTP superfamily is the largest class of all homeobox-bearing families and contains two subclasses: Hox-like (HOXL) and NK-like (NKL). The defining features of the HOXL subclass are its arginine (Arg) residue at position 5 and a glutamine (Gln) residue at position 50 (or lysine at 51) in the HD sequence (Table 1) [9]. The signature of the NKL subclass is an arginine residue at position 5 (or glutamine at position 6) and a glutamine at position 50 in the HD. The HOXL subclass has 53 members, and the NKL subclass contains 60 members. The NKL subclass is further divided into various subfamilies, including Nkx, EN, Ventx, Vax, Emx, NOTO, etc. These subfamilies contain several homeobox-bearing proteins similar to Ventx (in *Xenopus* and humans) and Vega (in zebrafish), Barx1, and Nanog. In *Xenopus*, there have been 251 identified homeobox proteins (Table 2) [11]. The Ventx family is one of the recently identified groups that contains six genes in *Xenopus* and three genes in zebrafish. These members play important roles in embryogenesis, such as axial patterning, germ-layer specification, cell positioning, proliferation and differentiation, and pluripotency. The HD of the NKL class contains three helix domains, of which domains 2 and 3 form a helix–turn–helix (HTH). The HD directly binds with 5′-TAA(T/A)T-3 cis-acting consensus elements in the 5′-flanking regions of target genes and modulates their transcription in a temporospatial and context-dependent manner [8,13,26]. Additionally, there is a well-conserved motif of K/Q/R(I/T/V)WF in the three helices of all the HD classes [8,9,27]. However, the functional role of this motif remains largely unclear. However, a role in determining the HD-binding specificity to their target DNA has been suggested [8].

## 2. Ventx Family in *Xenopus*

**Figure 3:**
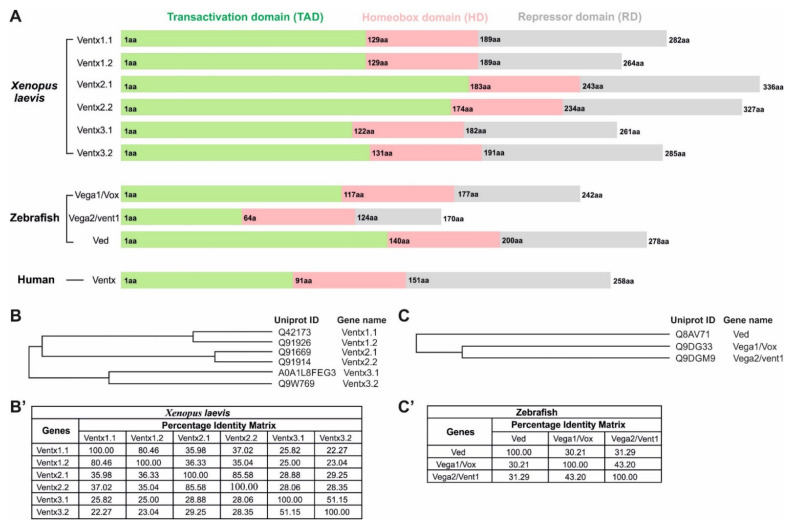
Schematic representations of Ventx domains in *Xenopus laevis*, zebrafish, and humans. (**A**) All Ventx members contain a transactivation domain (green), a highly conserved homeobox DNA-binding domain (HD, 60 aa, pink), and a repressor domain (grey), which inhibits the expression of dorsal-specific genes such as *gsc*, *chordin*, and *boz/dha*. The N-terminal domain (ND) of the Ventx family acts as a transactivation domain (TAD) to activate ventral-specific genes such as *bmp4*, *ventx1.1*, *ventx1.2*, *Ventx2.2*, and *Ventx2.2*. (**B**) An evolutionary phylogenetic tree of the Ventx gene cluster in *Xenopus laevis*. (**C**) An evolutionary phylogenetic tree of the *vega*/*ventx* gene cluster in zebrafish. (**B’**,**C’**) The percentage-identity matrix of Ventx family members in *Xenopus* and zebrafish.

In *Xenopus*, the Ventx family has six members, including Ventx1.1, Ventx1.2, Ventx2.1 Ventx2.2, Ventx3.1, and Ventx3.2 (Figure 3A). The phylogenetic tree of the Ventx family members in the *Xenopus* has indicated that Ventx1.1 and Ventx1.2 come from the same ancestor and showed 80.46% similarity in their protein sequences. However, Ventx3.1 and Ventx3.2 are the most differentiated members in the Ventx cluster and showed 51.15% similarity in their amino acid sequences in *Xenopus laevis* (Figure 3B,B’). All members of the Ventx family are positively regulated by BMP4/Smad1/5/8 signaling in the gastrulae, leading to ventral mesoderm, ectodermal formation, and embryonic ventralization [14,30,35]. Some members, such as Ventx1.1, Ventx2.1, and Ventx1.2, are also positively regulated by FGF/Xbra signaling in animal-cap explants, leading to mesodermal formation [13,23,35,70]. All members of Ventx are expressed in the ectoderm and ventral mesoderm of *Xenopus* embryos after mid-blastula transition (MBT) during gastrulation. The detailed functional and molecular mechanisms of these members are discussed below.

### 2.1. Ventx1.1

Ventx1.1 is also known as Ventx1.1a, Ventx1.1b, and posterior-ventral 1 (PV.1a). It consists of 282 aa with a molecular weight of approximately 32 kDa. The protein acts as an endogenous neural repressor, reaching a peak during gastrulation and then diminishing during the neurulation stage of *Xenopus* embryos. Moreover, high levels of *ventx1.1* expression have been reported in the ventral marginal zone and ectoderm in gastrulation stage embryos. A study demonstrated that BMP4/Smad1 signaling enhanced the expression of *ventx1.1* while blocking BMP4 signaling via a constitutively inactive, truncated form of BMP4 receptor I (DN-BR/tBR) that eliminated *ventx1.1* expression in uncommitted ectodermal explants of *Xenopus* embryos [71]. The ectopic expression of Ventx1.1 inhibits the ectoderm-to-neuroectodermal transition in intact embryos, leading to the inhibition of primary neurogenesis. Studies have shown that an elevated level of Ventx1.1 has led to embryonic ventralization and partially counteracted the effects of the dorsalizing agent lithium chloride (LiCl), a GSK3β inhibitor. It also counteracted the neurulation activity of Spemann-organizer-specific factors, including *chordin*, *noggin*, *gsc*, and *activin*, in *Xenopus* embryos [30,71], all of which led to the formation of the central nervous system. It is the C-terminal of Ventx1.1 that represses the dorsal and Spemann-organizer-specific genes in *Xenopus*, which results in a headless phenotype when ectopically expressed [33,72]. Moreover, the inhibition of Ventx1.1 by the engrailed–fused N-terminal region of Ventx1.1 (N-PV.1-EnR) in the ventral marginal zone (VMZ) induced a partial secondary axis. The inhibition of Ventx1.1 activity by an antimorphic form of Ventx1.1 (N-Ventx1.1-EnR) in VMZ triggered the expression of dorsal-specific genes, promoting secondary head formation.

A study showed that embryonic FGF (eFGF) inhibited BMP4-induced erythropoiesis in a Ventx1.1-dependent manner in the VMZ of *Xenopus* embryos [42]. eFGF/Ventx1.1 reduced the BMP4-induced erythropoietic markers, namely, *gata2* and *α-globin*, to inhibit erythropoiesis in *Xenopus* gastrulae. A recent study indicated that FGF signaling controlled the dorso–ventral mesodermal transition in *Xenopus* embryos [35]. In DMZ, the blocking of FGF signaling by the constitutively inactive form of FGF receptor (DNFR) or DN-Ras led the conversion of DMZ to VMZ in *Xenopus laevis*. Moreover, the inhibition of FGF signaling recovered the activin-reduced expression of ventral mesodermal markers in DMZ, including *ventx1.1*, *gata2*, and *α-globin*. Furthermore, FGF inhibition in DMZ induced *ventx1.1* expression to eliminate the expression of the organizer and dorsal-mesodermal-specific markers, including *chordin* and *noggin*, in the gastrulae [73,74,75]. These studies have suggested that Ventx1.1 is involved in ventral mesodermal formation and hematopoiesis in *Xenopus*. The ectopic expression of Ventx1.1 directly attenuated the mRNA levels and promoter activity of neuroectodermal specifiers, specifically *zic3* and *foxd4l1.1*, in *Xenopus laevis* embryos [76,77]. Ventx1.1 interacted within the promoter regions of neural and dorsal genes, such as *zic3*, *foxd4l1.1*, and *noggin*, in *Xenopus*. This reduction resulted in inhibiting the primary neurogenesis in *Xenopus* gastrulae. A recent study demonstrated that BMP4/Smad1 and FGF/Xbra cooperated synergistically to activate *ventx1.1* transcription, which led to the VMZ formation in *Xenopus* embryos [13]. BMP4/BMPR-I/II leads to the Smad1 (pS463/465) phosphorylation to catalyze the interaction of Smad1 with Xbra. This complex directly binds with the *ventx1.1*-promoter region and activates *ventx1.1* transcription in the VMZ. Furthermore, Smad1/Xbra-induced Ventx1.1 suppresses the DNBR-induced expression of early and late neural-specific markers, such as *foxd4l1.1*, *ncam*, *ngnr*, and *otx2*, consequently resulting in neural inhibition in the uncommitted explants of *Xenopus* embryos. A recent study documented that FoxD4l1.1 inhibited *ventx1.1* transcription directly as well as indirectly in the neuroectodermal region of *Xenopus* gastrulae. FoxD4l1.1 directly bonded within the proximal-promoter region of *ventx1.1* in *Xenopus* embryos. Apart from the direct inhibition of *ventx1.1* transcription, the ectopic expression of FoxD4l1.1 repressed Smad1/Xbra-induced synergistic transcription activation of *ventx1.1* in *Xenopus*. Additionally, FoxD4l1.1 physically interacted with Xbra and diminished the Xbra-binding within the 5′-upstream-promoter region to inhibit *ventx1.1* transcription. FoxD4l1.1 also reduced C-terminal phosphorylation of Smad1 to arrest nuclear localization of Smad1, which then repressed Smad1-induced *ventx1.1* transcription and led to neuroectodermal formation in *Xenopus* gastrulae [78,79]. A study reported that an elevated level of Ventx1.1 also repressed VMZ markers, including *ventx1.1*, *ventx1.2*, and *ventx2.1*, without affecting the *bmp4* expression during gastrulation in *Xenopus* embryos [80]. Moreover, Ventx1.1 reduced the *xcad2*-induced (a transcriptional activator of ventral signaling [81]) expression of ventral mesoderm-specific markers in animal-cap explants. Ventx1.1 directly bonded within its own promoter region and negatively regulated its own transcription in gastrulae. Interestingly, Ventx1.1 and Xcad2 share the same cis-acting Ventx1.1-binding-response elements (VRE) to regulate VMZ-specific genes in an opposite manner. Ventx1.1 attenuated ventral mesodermal markers while Xcad2 promoted the expression of these markers in animal-cap explants [80]. The concomitant expression of Ventx1.1 and Xcad2 competed and reduced the direct binding of Xcad2 within the 5′-upstream regions of *ventx1.1* to inhibit the Xcad2-induced expression of the Ventx family genes, including *ventx1.1*. The Ventx1.1-mediated inhibition of its own expression in a recurrent inhibitory loop suggests that Ventx1.1 controls its own concentration in the ventral mesoderm and the ectoderm in order to guide the orderly dorsoventral patterning during development. These studies have shown that Ventx1.1 is critical in axial patterning, hematopoiesis, and VMZ formation in *Xenopus*.

### 2.2. Ventx1.2/Xvent1

Ventx1.2 is also known as Vent1 in *Xenopus*. Ventx1.2, composed of 264 aa and containing a 60-aa-long DNA-binding domain (DBD) called homeobox, binds within the 5′-updtream region of its targets. The molecular weight of Ventx1.2 is approximately 30 kDa (Figure 3A). Being similar to ventx1.1, Ventx1.2 is also a homeobox transcription factor and an endogenous neural repressor. BMP4/Smad1 also led to the expression of *ventx1.2*, which was differentially expressed in the ventral mesodermal zone, ectoderm, and VBIs of *Xenopus* [46,82]. Ventx1.2 supressed dorsal-lip-specific and Spemann-organizer-specific genes, including *xfd-1* (a neural specifier and anterior–posterior neural-patterning marker) and *gsc*, inhibiting head formation and embryonic ventralization [43]. This inhibition was caused by the direct binding of Ventx1.2 within the 5′-upstream region of *xfd-1* and the blocking of *xfd-1* transcription in *Xenopus* embryos. Moreover, the inhibition of Ventx1.2 has promoted the transcription of *xfd-1* to trigger neural induction in uncommitted explants of *Xenopus* embryos.

A recent study indicated that the loss of *ventx1.2* in VMZ resulted in a partial secondary axial induction and embryonic dorsalization [47]. Moreover, the loss of Ventx1.2 enhanced the expression of anterior neural specifiers, including *xag1* (cement gland marker), *en-2* (anterior marker), and *ncam* (pan-neural marker) in uncommitted animal-cap explants. Interestingly, an elevated level of Ventx1.2 also induced stem-cell-leukemia (*scl*) expression in uncommitted explants of *Xenopus* gastrulae. Scl is a hematopoiesis marker that synergistically triggers the hematopoietic cell differentiation with GATA1 and LMO2 (yolk-sac-erythropoiesis marker) in *Xenopus* [82]. In summary, Ventx1.2 may actively participate in hematopoiesis, cell fate determination, and embryonic ventralization in *Xenopus*.

### 2.3. Ventx2.1

Other names for Ventx2.1 include Vox, Xvent2, and Ventx2.1a/b. Ventx2.1 is the longest member of the Ventx transcriptional repressor family at 336 aa in length, and it has a molecular weight of approximately 38 kDa (Figure 3A). Ventx2.1 contains three distinct domains: the N-terminal transactivation domain (TAD), the DNA-binding homeobox domain (DBD), and the C-terminal repressor domain (RD). The expression of Ventx2.1 is primarily reported during blastulation after the mid-blastula transition (MBT) and leads to the development of the ventral mesodermal and ectodermal regions of *Xenopus* embryos. Studies have reported that BMP4/Smad1 directly induced the expression of *ventx2.1* in VMZ and ectoderm in *Xenopus laevis* [24,82]. The TAD of Ventx2.1 activates the transcription of ventral and ectodermal specifiers, namely, *ventx1.1*, *bmp4*, and itself, in a positive feedback loop during the embryonic development of *Xenopus* embryos [14,24,25]. Additionally, Ventx2.1 directly binds to the 5′-upstream regions of *bmp4* and *ventx1.1* and activates their transcription in embryos’ gastrulae, leading to embryonic ventralization.

Studies have reported that a morpholino-based knockdown of *ventx2.1* caused a partial secondary axis and expanded the Spemann-organizer region in *Xenopus* embryos [24,47,83]. The knockdown of *ventx2.1* in the VMZ region induced the expression of Spemann-organizer specifiers, including *noggin*, *chordin*, and *gsc*, in *Xenopus* embryos. Moreover, FGF/Xbra also bonded within the 5′-upstream regions of *ventx2.1* in *Xenopus* gastrulae. The FGF/Xbra pathway positively induced *ventx2.1* transcription to trigger ventral mesodermal and ectodermal formation in *Xenopus* [13]. A study has also reported that the physical interaction of Ventx2.1 and GATA2 induced the expression of *bmp4* in an auto-positive feedback loop to augment *ventx1.2* expression, which led to embryonic ventralization and ventral mesodermal formation in *Xenopus* embryos [22]. Lee et al. (2011) showed that Ventx2.1 alone could activate *ventx1.1* transcription after the direct binding of Ventx2.1 within the 5′-upstream regions of *ventx1.1* during gastrulation in *Xenopus* [14]. The RD of Ventx2.1 formed a complex with the MH1 domain of Smad1 in VMZ to synergistically activate *ventx2.1* transcription. This activation was synergized in a positive feedback loop, which led to embryonic ventralization and mesodermal formation [12]. Recently, it was documented that the concomitant expression of Ventx2.1 and Ventx1.2 repressed the expression of early neuroectodermal progenitor markers, including *sox3*, *sox2*, *geminin*, in *Xenopus* embryos [84]. This repression might be caused by the repressor domains (RDs) of Ventx2.1 and Ventx1.2 in *Xenopus*, inhibiting the head and neural formation. It has been shown that activin could induce the expression of *ventx2.1* in uncommitted explants [85]. However, the function of the activin-mediated induction of *ventx2.1* was unknown until recently. Ventx2.1 promoted BMP4 signaling in a positive feedback loop to support dorsoventral axial patterning and germ-layer specification.

### 2.4. Ventx2.2/Xom/Xbr-1

Ventx2.2 is also known as Xvent-2 and Xom-A. The structural details of Ventx2.2 are available in Figure 3A. Being similar to Ventx2.1, Ventx2.2 comprises three domains: the N-terminal transactivation domain, the middle DNA-binding domain (DBD), and the C-terminal repressor domain. BMP4/Smad1 has also induced *ventx2.2* expression in the VMZ and the ectodermal region of *Xenopus laevis* [26]. The ectopic expression of Ventx2.2 promoted embryonic ventralization by directly inhibiting the expression of *gsc* (*gsc* is an endogenous transcriptional inhibitor of ventral-specific genes). Ventx2.2 physically interacted within the 5′-upstream regions of *gsc*, leading to the transcriptional inhibition of *gsc* in *Xenopus* embryos. A previous study showed that Ventx2.2 had been phosphorylated in a GSK3β-like domain (DpSGYEpS) and was degraded in a GSK3β-independent manner in *Xenopus* gastrulae [86]. The phosphorylated Ventx2.2 interacted with E3 ubiquitin ligase, SCF-β^TrCP^, to degrade Ventx2.2 during early gastrulation in *Xenopus* embryos.

Moreover, the dorsal injection of a phospho-dead mutant of Ventx2.2 (i.e., Xom-2SA) repressed the dorsally expressed genes effectively during the gastrulation of *Xenopus* embryos. This repression of dorsal genes then led to ventralization and a headless phenotype. A recent study has reported that the N-terminal (TAD) of Ventx2.2 interacted with LEF1/TCFs, a high mobility group box-containing transcription factor, and transactivated the LEF1/TCFs-mediated transcription of ventrally expressed genes in *Xenopus* embryos [87]. In addition, the N-terminal-deleted Ventx2.2 failed to induce the TAD-mediated transactivation of ventral-specific genes, while retaining its repressor activity to suppress *gsc* transcription in *Xenopus* animal-cap explants. Additionally, the TAD-lacking Ventx2.2 failed to activate *bmp4* expression and resulted in an apoptosis-like response in *Xenopus* embryos [88,89]. Another recent study showed that Ventx2.2 balanced the concentrations of β-cat and GSK3β in the dorsal and ventral regions, respectively, leading to dorsoventral axial patterning during the embryonic development of *Xenopus* [90]. Ventx2.2 triggered the β-cat proteolysis in a GSK3β-dependent manner in the ventral region. Ventx2.2 induced *gsk3β* expression while GSK3β catalyzed the phosphorylation and degradation of β-cat in the VMZ to initiate the orderly patterning of the dorsoventral axis during embryonic development. These studies indicated that Ventx2.2 acts as a vital player during dorsoventral axial patterning and neural crest differentiation in *Xenopus*.

### 2.5. Ventx2.2 Maintains Cellular Pluripotency in Xenopus Similarly to Nanog in Other Animals

*Xenopus* lacks the Nanog gene, a pluripotent and proliferative marker in humans, mice, and zebrafish [91]. Nanog is a homeobox transcription factor and is expressed in mammalian pluripotent cells and developing germ cells. Nanog expression is considered as a hallmark of pluripotency in vivo as well as in vitro [92]. The deletion of Nanog in mammalian cells causes embryonic lethality. The constitutive expression of Nanog has resulted in the self-renewal of embryonic stem cells (ESCs) and sustained their uncommitted state [92]. Additionally, Nanog-null cells showed multi-lineage differentiation both in fetal and adult mice. Multiple lines of evidence have suggested that Ventx2.2 may play a similar role to Nanog in the development of *Xenopus*. The sequence homologies between Ventx2.2 and Nanog within the BarH subclass of the NKL family indicated that they may have descended from the same ancestor. Both Ventx2.2 and Nanog contain the BarH-specific threonine (Thr) residue and the conserved serine (Ser) residue outside the HD, which are absent in all other BarH-subclass proteins. In addition, the temporal expressions of *Xenopus ventx2.2* and zebrafish Nanog (zNanog) are very similar, and overexpression of either results in the same phenotype [91]. Furthermore, both Ventx2.2 and Nanog dimerized, directly interacted with Oct25, and inhibited the expression of dorsalizing factors *gsc* and *chordin* in zebrafish. Ventx2.2 not only led to ventral mesodermal differentiation and embryonic ventralization but also determined the pluripotency in embryonic cells, such as those in animal caps. A recent study proposed that Ventx2.2 acts as a Nanog ortholog to maintain cellular pluripotency in *Xenopus* [48]. In *Xenopus*, the overexpression of murine Nanog (mNanog) has led to dorsal mesodermal formation by suppressing the effect of BMP4 in uncommitted ectodermal cells [85]. In addition, overexpressed mNanog induced the expression of *chordin* and *gsc* (endogenous inhibitors of BMP4 signaling), causing a secondary axis in *Xenopus*. mNanog inhibited the expression of *Ventx2.2* (a positive regulator of BMP4 signaling), suppressed BMP4 signaling, and retained the expression of dorsal-specific markers, including *chordin*, *Xnot*, and *gsc*, to trigger dorsal mesodermal formation in the uncommitted ectoderm of *Xenopus* embryos [85]. A recent study showed that the antisense-morpholino-based combined inactivation of Ventx1 and Ventx2 (Ventx1/2) reduced the expression of the multipotency marker *oct91* and premature differentiation in *Xenopus* blastulae [48]. The expression of *oct91* is required to maintain the pluripotency and premature cell differentiation during *Xenopus* blastulae. The concomitant overexpression of Ventx1/2 obstructed the lineage commitment of a dorsoventral fate; and the overexpression of Ventx1/2 and mNanog reduced the expression of germ cell specification, separately, in a similar manner [48]. A knockdown of Ventx1/2 repressed pluripotency markers and led to premature cell commitments during blastulae [48,49]. Ventx1/2 notably reduced the expression levels of organizer-specific genes, including *gsc*, *hhex*, and *Xnot*, to inhibit the dorsal mesodermal differentiation in animal-cap explants, similarly to mNanog. In addition, similarly to the dorsal mesoderm, the concomitant expression of Ventx1/2 mRNA also suppressed the expression of the ventral mesodermal (*bmp4*) and early ectodermal markers (*lim5* and *foxi1a*). Conversely, Ventx1/2 and mNanog significantly reduced the expression of genes involved in epidermis (*tfap2a*), axial (*xbra*), and paraxial mesodermal (*myf5*) in *Xenopus*. These results indicate that Ventx1/2 inhibits the dorsoventral fate commitment in *Xenopus*, similar to mNanog. Strikingly, the ectopic expression of mNanog mediated the Ventx1/2 deficiency in *Xenopus* embryos, leading to embryonic dorsalization. These studies showed that Ventx1/2 acted as a Nanog ortholog in *Xenopus* by maintaining pluripotency in *Xenopus* blastulae to delay the multiple-cell lineage commitment and differentiation, and inhibited the onset of dorsoventral axial patterning in *Xenopus* [48]. Recently, a study indicated that MEK1 (Map2k1), an upstream kinase of the MAPK family, phosphorylated Ventx2.2 in the PEST-like destruction motif (a region rich in proline (Pro), glutamate (Glu), Serine (Ser), and threonine (Thr)) and destabilized Ventx2.2, leading to uneven dorsoventral gradients of Ventx2.2 during the embryonic development of *Xenopus* [49]. By destabilizing Ventx2.2, MEK1 promoted multiple-cell lineage commitments and differentiation to initiate gastrulation in *Xenopus* embryos. An elevated level of Ventx2.2 blocked neuroectodermal differentiation and promoted the inhibition of neural progenitor markers, including *sox2* and *sox3*, in *Xenopus* embryos [84]. A recent study demonstrated that the ectopic expression of Ventx2.2 regulated the reprogramming of uncommitted ectoderm towards an early neural-crest cell identity in *Xenopus*, leading to ectomesenchyme differentiation [93]. Ventx2.2 helped to maintain a population of multipotent progenitor cells in the pre-existing sensory neurons and pigment neural borders and led to skeletogenic neural-crest formation. Ventx2.2 also induced the expression of neural-crest specifiers at the neural plate border (*foxd3*, *twist1*, *snail1/2*, and *soxE*, a pan-vertebrate neural crest marker) and stem-cell marker (*pou5f3.1/oct4*), along with other reprogramming-associated genes (*chd7*, *tert*, *tet3*, *kdm4a*, and *smarc4a*) in *Xenopus* embryos. Additionally, the morpholino-based knockdown of ventx2.2 reduced the expression of neural-crest- and multipotency-specific markers and caused severe defects in the cranial neural crest (CNC) and ectomesenchyme formation in *Xenopus*. In summary, Ventx2.2 has the potential to regulate multiple steps of the neural-crest-gene regulatory network in *Xenopus*.

### 2.6. Ventx2.2 Leads to Ectomesenchyme Proliferation and Mandibular Cartilage Development in Xenopus as Barx1 Does in Rodents and Fruit Flies

As we have discussed above, Ventx synteny was absent in rodents and the fruit fly, *D. melanogaster*. The genes closely related to Ventx2.2 in mice (BarH1 and BarX1) and *Drosophila* (BarH1) belong to the Bar family of the homeobox. Both the Ventx and the Bar families share a rare amino acid substitution (Thr instead of Val/Ile) at position 47 of the third helix of the HD. In *Xenopus*, Ladher et al. (1996) and Papalopulu et al. (1996) independently isolated *ventx2.2* and named it “Xom” for its similarity to the *Om(1D) Drosophila annasae* homologue of *D. melanogaster BarH1* and *Xenopus* Bar-related *(Xbr-1)* [27,28]. Xom and Xbr-1 share similarities in sequence (approximately 55%) and expression patterns to the HD of *Drosophila BarH1/Om(1D)*. Similarly to Ventx2.2, BarX1 promoted ectomesenchyme development and neural-crest cell migration, leading to craniofacial skeleton formation in vertebrates. As we discussed above, Ventx2.2 is also involved in neural-crest cell migration and ectomesenchyme formation [93]. Neural-crest cells originate from four major parts of the neural axis: cranial, cardiac, Vagal, and trunk [94]. These cells further differentiate into various types of cells, including neurons, connective tissue cells, myocytes, enteric ganglia, melanocytes, sympathetic ganglia, etc. Studies have shown that Barx1 was expressed in pharyngeal dentition and required for tooth development in mice and zebrafish [95,96,97]. A recent study demonstrated that Barx1 led to tooth and musculoskeletal attachment but not tooth initiation and morphogenesis in zebrafish [97]. In Barx1 mutants, the intermandibular muscles were disorganized, failed to attach to Meckel’s cartilage, and invaded the cartilage gaps instead (Meckel’s cartilage gap is a hallmark of jaw-joint identity). In addition, sternohyal muscles were shorter, and hyohyal muscles were reversed in their orientation. These muscles were specified clearly but failed to attach to their skeletal scaffolds, resulting in craniofacial defects. The repression of Barx1 at the mandibular arch is required for the development of the mandibular moveable joint, and its overexpression is needed for chondrocyte differentiation to form cartilage and support the craniofacial skeletal’s formation. BMP4 induced the expression of *barx1* in the pharyngeal arch region of zebrafish and led to chondrocyte differentiation and condensation [98]. The loss of Barx1 resulted in the inhibition of chondrocyte differentiation and a reduction in the odontogenic marker, *dlx2b*, which is needed for chondrogenesis in zebrafish. In addition, the loss of Barx1 regulated jaw-joint patterning by inducing *gdf5* (a joint and cartilage marker) and *chordin* (a jaw-joint-patterning marker) expression, promoting the development of the craniofacial skeleton. These studies indicated that Barx1 is a downstream target of BMP4 signaling, like Ventx2.2, and plays a role in neural-crest cell migration and ectomesenchyme proliferation in zebrafish.

Ventx2.2/Xom is also known as a canonical Wnt/β-cat inhibitor in human and *Xenopus* [87,99]. Ventx2.2/Xom physically interacted with LEF/TCF and blocked its interaction with β-cat, promoting ventral gene expression in *Xenopus*. LEF/TCF and Ventx2.2/Xom diminished the β-cat level in cells and inhibited the dorsalizing effect of Wnt/β-cat during embryonic development. Similarly, Barx1 inhibited Wnt signaling in a sFRP1/2-dependent (a secreted-Wnt antagonist) manner in mice [100]. Wnt/β-cat led to gastric endodermal differentiation instead of epithelial differentiation. The Barx-1-mediated inhibition of Wnt/β-cat promoted stomach-specific epithelial differentiation and suppressed endodermal differentiation in mice. A recent study showed that the loss of Barx1 caused defects in the positioning and expansion of the spleen (a mesothelium-lining-originated organ) in a WT1-dependent manner [101]. The loss of Barx1 in mice reduced the expression of *WT1* (a mesothelium marker that is important in spleen morphogenesis). Therefore, Barx1 is critical for spleen development and its positioning during gastrointestinal organogenesis. In conclusion, both Ventx2.2 and Barx1 are highly involved in neural-crest migration, ectomesenchyme and chondrocyte differentiation, and organogenesis.

### 2.7. Ventx3.1

Ventx3.1 is also known as Vent-3 and consists of 261 aa with a molecular weight of approximately 30 kDa (Figure 3A). The functions of Ventx3.1 have not yet been studied in *Xenopus laevis*.

### 2.8. Ventx3.2/Vex1

Ventx3.2, also known as Vex-1, is one of least studied members of the Ventx family in *Xenopus*. Ventx3.2 is composed of 285 aa and its molecular weight is approximately 32 kDa (Figure 3A). Ventx3.2 is one of the direct downstream targets of BMP4/Smad1 signaling [37,102]. It is expressed in VMZ and the ectodermal region throughout gastrulation.

**Figure 4:**
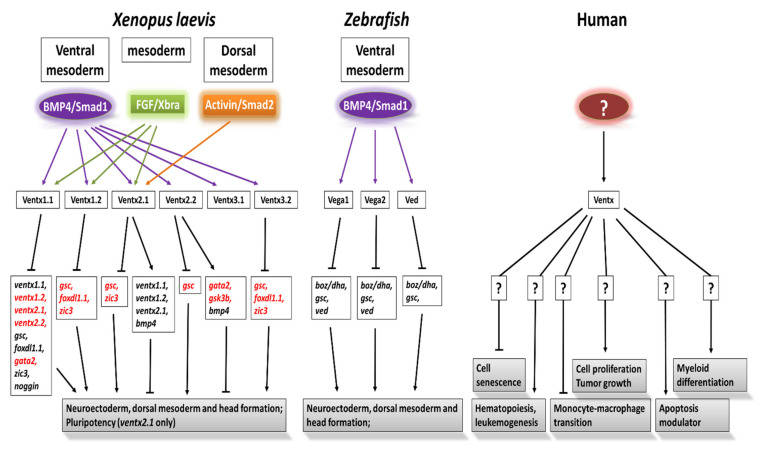
Upstream and downstream regulatory pathways for Ventx members during *Xenopus laevis* and zebrafish embryonic development and the potential functions of Ventx in human hematopoiesis, cell differentiation, and leukemogenesis. Lines with arrows indicate promoting and those with bars suppressing (functions of) the targets. Indirect targets of Ventx family members are in red. Sign (?) indicates unknown factors.

Overexpressed Ventx3.2 led to embryonic ventralization in a dose-dependent manner and remedied the effect of DNBR-inhibited BMP4 in *Xenopus* embryos [102]. Moreover, the overexpression of Ventx3.2 directly inhibited the expression of dorsal- and anterior-specific neural genes, including *gsc* and *otx2*, in animal-cap explants. Antisense RNA and the morpholino-mediated inhibition of *ventx3.2* in VMZ resulted in embryo dorsalization and secondary axial formation by enhancing the expression of *gsc* and *chordin* in the VMZ of *Xenopus*. These studies indicated that Ventx3.2 functioned similarly to most other Ventx family members and promoted ventral mesodermal formation and neural inhibition in *Xenopus laevis*. The Ventx family signaling crosstalks that are regulated by BMP4/Smad1, FGF/Xbra, and activin are summarized in Figure 4.

## 3. Vega Family in Zebrafish

The Vega family in zebrafish is also known as the Vent family. It is the equivalent to the Ventx family in *Xenopus laevis*, as they have identical physiological roles. The Vega family synteny in zebrafish are located on two different chromosomes: Vega1 and Vega2 on chromosome 13; and Ved on chromosome 10 (Table 3). The evolutionary phylogenetic tree of the Vega/Ventx family cluster in zebrafish indicates that Vega1 and Vega2 descended from a common ancestor and that Ved was once a member of the Vega family (Figure 3C,C’). During evolution, Ved might have shifted to chromosome 13, since Vega1/2 and Ved follow the root of common ancestors in zebrafish. Similarl7 to the Ventx family in *Xenopus*, BMP4/Smad1/5 signaling upregulated Vega family expression in zebrafish, leading to dorsoventral axial patterning and VMZ formation [21]. The functions of the Vega family in zebrafish are described below.

### 3.1. Vega1/Vox

Vega1 is one of the first identified members of the Vega family in zebrafish. Vega1 consists of 242 aa, including a 66-aa-long C-terminal repressor domain, and its molecular weight is approximately 28 kDa (Figure 3A). The transcription of *vega1* is activated in the mid-blastula transition (MBT) during blastulation, and it is downregulated dorsally before gastrulation onset. The expression of *vega1* has been reported in the VMZ region, and elevated levels have been shown to inhibit the expression of a dorsal-specific gene named *bozozok/dharma* (*boz/dha*), which is required for organizer formation and embryo dorsalization), *chordin* (*dino*), and *gsc* genes in zebrafish [34,44]. The reduced levels of dorsal-specific genes caused a headless phenotype and organizer-expansion inhibition, similarly to Ventx1.1 in *Xenopus*. Additionally, the dominant-negative mutant of Vega1 mitigated the effects of *gsc* and *boz/dha* in VMZ and led to embryonic dorsalization and secondary axial formation in zebrafish. Therefore, Vega1 is required for dorsoventral axial patterning in zebrafish.

### 3.2. Vega2/Vent1/Vent

Vega2 is also known as a ventral-expressed homeobox (Figure 3A). The transcription of *vega2* is found in the VMZ during blastulation, and it is downregulated dorsally, similarly to Vega1. The expression of *vega2* partially overlapped with a dorsal-specific gene, *gsc*, in zebrafish embryos. Similarly to the Ventx family members, the expression of *vega2* was also positively regulated by BMP4/Smad1 signaling in the VMZ region of zebrafish embryos. Overexpressing Vega2 in DMZ inhibited *gsc* and caused a headless phenotype during the embryonic development of zebrafish. The interaction of Vega2 and Vega1 synergistically inhibited the transcription of *gsc*, causing ventralization and VMZ formation in zebrafish [44,45]. Additionally, the dominant-negative mutant of Vega2 induced *gsc* expression in VMZ and resulted in embryonic dorsalization in zebrafish. Vega2 thus supported dorsoventral axial patterning and germ-layer specification in zebrafish. These studies also suggested that vega1 and Vega2 could possibly act as negative regulators for organizer expansion and head formation in zebrafish.

### 3.3. Ved

Unlike Vega1 and Vega2, Ved is located on chromosome 10 in zebrafish (Table 3). Ved is the longest member of the Vega family, being 278 aa in length, and it has a molecular weight of approximately 31 kDa (Figure 3A). Ved is also a transcription repressor, and it expresses ventrally in zebrafish. *Ved* has been reported as a direct target of Boz/Dha in zebrafish. A recent study has shown that BMP4 signaling ventrally controlled the expression of Ved, and the dorsal, combined inhibition of *dino* and *gsc* induced the expression of *ved* in the DMZ of zebrafish embryos, leading to embryonic ventralization, a shortened anterior structure, and a headless phenotype [103]. A study showed that the combined morpholino-based knockdown of *ved*, *vega1*, and *vega2* in zebrafish embryos enhanced the expression of *boz/dha*, *gsc*, and *dino*, resulting in an organizer-center expansion [38].

These studies collectively indicated that the Vega family works as one of the downstream targets of BMP4 signaling and triggers embryonic ventralization and VMZ formation. Furthermore, the inhibition of the Vega family resulted in the organizer-region expansion and embryonic dorsalization. Taken together, the studies indicated that the entire Vega family plays an essential role in the dorsoventral axial patterning of zebrafish embryos. The functions of the Vega family are summarized in Figure 4.

## 4. Human Ventx (VENT-like Homeobox Protein 2)

Ventx might be the only reported active member of the Ventx family in humans (Figure 3A). It is also known as VENTX2 and HPX42B. Human Ventx showed 39%–67.66% similarity to Ventx family members in *Xenopus* and zebrafish (Table 3) [17]. The ectopic expression of human Ventx in zebrafish embryos caused the loss of the anterior structure, resulting in the failure of notochord formation. In addition, overexpressed Ventx also leads to embryonic ventralization in *Xenopus* and zebrafish. Similarly to the Ventx family in *Xenopus* and zebrafish, the expression of human Ventx was also led by BMP4 signaling [108]. Human Ventx works downstream of BMP4/Smad1 signaling and causes embryonic ventralization in *Xenopus* and zebrafish. A study reported that Ventx was aberrantly expressed in CD34^+^ acute myeloid leukemia (AML), and its maximum expression was found in CD33^+^ myeloid cells but not in normal CD34^+^/CD38^−^ leukemic cells [18]. In addition, the abnormal expression of Ventx in normal CD34^+^ cells could disturb the development of hematopoietic cells, leading to myeloid cell differentiation rather than lymphoid generation, both in vivo and in vitro. The shRNA-mediated knockdown of *Ventx* resulted in the failure of AML cell proliferation [18]. These studies indicated that Ventx may play a vital role in postnatal hematopoiesis and malignant myelopoiesis.

Ventx led to macrophage differentiation, depending on the cytokines involved, such as CSFs and IL-3. The inhibition of Ventx in primary monocytes suppressed monocyte-to-macrophage differentiation, suggesting that Ventx plays a significant role in macrophage differentiation and the ensuing activation of cytokines [51]. A recent study has shown that Ventx was dramatically reduced in tumor-associated macrophages (TAMs), which are critical components of the tumor microenvironment (TME). The suppression of the immune system in TME was one of the key hurdles in developing effective immunotherapies to treat tumors [109]. In addition, Ventx potentiated the cell growth and terminal differentiation of hematopoietic and immune cells in isolated samples of cancer patients. Moreover, Ventx regulated TAM plasticity, resulting in the M1-like phenotype of TAMs. Ventx suppressed the immune system in the TME, inhibited regulatory T-cell (Treg) differentiation, and promoted CD8 tumor-infiltrating lymphocytes [109]. In addition, Ventx modulated TAM functions by inhibiting the immune suppression of TAM plasticity in the TME during tumorigenesis in colon-cancer models. The involvement of Ventx in macrophage differentiation and the suppression of TAM plasticity in TME indicates that Ventx could be a novel agent for targeting tumorigenesis. Recently, Le et al. (2020) showed that the expression level of Ventx was notably decreased in the pancreatic ductal adenocarcinoma-(PDA)-associated macrophages (TAMs) collected from verified human patients [110]. The study showed that Ventx was necessary for regulating the phagocytosis, and the restored level of Ventx triggered phagocytosis by regulating the expression of Toll-like receptors 2/9, MyD88, and Mapk38 in PDA TAMs. Furthermore, the ectopic expression of Ventx inhibited the SHP1/2 phosphorylation level (the downstream molecules of SIRPa-CD47 signaling) to trigger phagocytosis. Ventx also induced phosphorylation focal adhesion kinase (FAK), leading to phagocytosis. Moreover, the elevated level of Ventx promoted the M1-like TAM phenotypes over M2-like TAM phenotypes to provide immunity against PDA by promoting the CD8 T-cell population over the Treg cell population in the PDA-associated tumor microenvironment. A study has shown that the M1/M2 and CD8/Treg ratio was associated with PDA prognosis [111], and the increased population of M1-like TAMs promoted immunity against pancreatic cancers. These findings indicate that Ventx could be a valuable candidate to improve PDA prognosis through the promotion of M1-like and CD8 populations and phagocytosis in PDA.

An elevated level of Ventx was reported in immature human hematopoietic cells. The knockdown of the Ventx expression significantly expanded the population of human stem cells and multipotent progenitor cells (HSC/MPP) and promoted the survival of HSC, ex vivo [112]. The suppression of *Ventx* in grafted HSC/MPP cells also increased the engraftment potential of HSC/MPP in NOD/SCID/IL2Rγ2^null^ murine models. This suggested that Ventx could be a potent target for the application of HSC/MPP-based therapy. Recently, a study demonstrated that the expression of *Ventx* robustly increased in normal myeloid cells and in AML patients [32]. The overexpressed Ventx in myeloid cells reduced the expression of myeloid-terminal-differentiation-related genes in healthy CD34^+^ stem and progenitor cells. Moreover, the transplantation of retrovirally-engineered Ventx-expressed bone marrow progenitor cells increased the population of primitive erythroid cells to trigger acute erythroid leukemia in transplanted mice. Furthermore, the concomitant expression of AML1-ETO (AML1-ETO, a fusion product of the chromosomal translocation t(8;21)(q22;q22)) usually occurs in acute myeloid leukemia (AML) with granulocytic differentiation, FAB M2 subtype [113], and Ventx induced the expression of the erythroid markers, leading to AML in all transplanted mice. In addition, high levels of Ventx in human erythroleukemia patients significantly inhibited cell proliferation and tumor growth. However, Ventx acted as a tumor activator and triggered leukemogenesis, but some studies demonstrated its tumor-suppressant role in humans. A study reported its reduced expression in peripheral blood of chronic lymphocytic leukemia (CLL) cells and that it acted as a tumor suppressant [99]. Ventx interacted with LEF/TCFs and inhibited the canonical Wnt/β-cat signaling (a positive regulator of cell proliferation) in CLL cells. Ventx and LEF/TCF interaction abrogates β-cat and LEF/TCFs complex formation and suppresss the proliferation-marker cyclin D1’s expression in CLL cells. Ventx-LEF/TCF interaction reduced the LEF/TCF-targeted oncogene, cyclin D1, to inhibit cyclin D1-mediated cell proliferation. The reduced level of Ventx in CLL cells promoted Wnt/β-cat and LEF/TCFs interaction, and in turn, induced cyclin D1 expression, leading to cell proliferation and cancer progression. Recently, it was reported that the differential methylation level of Ventx was significantly implicated in sarcoma progression in humans [114]. A genome-wide methylation-profiling study in metastatic and recurrent sarcomas showed that Ventx was hypermethylated in metastasis pleomorphic rhabdomyosarcoma (Ple-RMS, a rare, aggressive sarcoma with poor prognosis), as compared to primary Ple-RMS. This study hypothesized that due to its involvement in tumor microenvironment and acting as an Wnt antagonist, Ventx may have strong therapeutic potential for sarcomas associated with immune cell content. A study showed that Ventx might also act as a negative regulator of tumor progression. Ventx activated both p16^ink4a^-Rb and p53–p21 tumor-suppression pathways [54]. Overexpressing Ventx led to a cell-senescence-like phenotype, followed by an irreversible cell-cycle arrest. Strikingly, the RNAi-mediated inhibition of *Ventx* enhanced cell senescence and ameliorated the camptothecin (CPT) resistance in lymphocytic leukemia cells. Ventx-associated chemotherapeutic resistance and its involvement in the cell-senescence process suggested that Ventx could be a novel, potential target for cancer prevention. A recent study confirmed the anticancer role of Ventx and showed that Ventx triggered apoptosis in a p53-independent manner in cancer cells (HCT116 p53KO) but not in healthy cells, in vitro [115]. In addition, Ventx activated the executioner caspase-3 in p53^−/−^ knockout cells, leading to the suppression of tumor growth.

Furthermore, the treatment of doxorubicin increased the level of Ventx in both p53 (WT) and p53^−/−^ knockout cells. Ventx’s involvement in hematopoiesis, leukemogenesis, cell senescence, and macrophage differentiation is illustrated in Figure 4. These studies suggested a tumor-suppressant function of Ventx in both p53^+/+^ and p53^−/−^ cells, and Ventx might be a novel therapeutic target for certain human cancers, working in a p53-independent manner. In summary, Ventx is deeply involved in hematopoiesis and leukemogenesis in humans, and it could be a future therapeutic target for combating tumorigenesis.

### Nanog (Ventx-like) Is Considered an Oncogene in Humans

Nanog is a well-established stem-cell pluripotent marker and homeobox transcription factor which creates stem-cell-like properties in cancer cells [92]. Earlier, we discussed the functional and homologic similarities between Ventx2.2 and Nanog and their roles in maintaining cell pluripotency and inhibiting differentiation. Cancer stem cells (CSCs) and normal stem cells can self-differentiate and self-renew. CSCs are immortal in nature, persistent in tumors, and provide nourishment for tumor maintenance, metastasize, and relapses [116]. Similarly to Ventx, aberrant expression of Nanog was detected in a small subset of AML patients and an AML cell line, Namo-1 (serves as a model for AML) [117]. Loss and gain of function studies have shown that Ventx, KLF4, Myb, and anti-apoptosis-factor MIR17HG are Nanog-targeted genes [116]. These factors are widely involved in generating oncogenic properties and leading to leukemogenesis in humans. Nanog inhibited p53 in a Gli-MDM2-dependent manner in glioma tumors [118,119]. Nanog induced the expression of Gli1/2 (glioma-associated oncogenes) that is required for MDM2 activation, and inhibited p53 activity. The tumor suppressant p53 directly reduced the promoter activity of *Nanog* (p53 is a direct inhibitor of Nanog). Nanog and p53 mutually inhibit each other expression in glioma tumors (for a review, see Grubelnik et al., 2020). However, p53 promoted Notch1 expression in early-stage oral squamous cell carcinoma. Notch1 is a positive regulator of Nanog [120]. The Nanog-mediated inhibition of p53 indicated that Nanog was involved in cancer cell proliferation and tumor development in humans. Additionally, the p53-mediated inhibition of Nanog suggested that Nanog had oncogenic abilities and may promote the stem-cell-like nature of cancer cells, leading to cancer self-differentiation and self-renewal [116,120]. Meanwhile, the p53-mediated activation of Nanog in a Notch1-dependent manner indicated the importance of Nanog during embryonic development.

Although, Ventx synteny is absent in rodent, but a recent study has been demonstrated that Nanog acts like a Ventx ortholog in rodents [121]. It showed that IGF2/IGF1R/Nanog signaling cascade regulates the proliferation of leukemia stem cells (LSCs) in mice. The overexpressed level of Nanog has been reported in CD34+ acute myeloid leukemia (AML) cells of patients and LSCs cells. Additionally, the knockdown of Nanog notably inhibits cell proliferation and induces cell cycle arrest and apoptosis. The si-mRNA-mediated silencing of Nanog suppresses the leukemogenesis of LSCs in mice. This report indicates that Nanog acts as a downstream target of IGF2/IGF1R signaling pathways that might positively regulate the Nanog transcription in LSCs. The overexpressed Nanog rescue colony formation ability of LSCs treated with IGF1R inhibitor (picropodophyllin). The knockdown of Nanog diminishes the colony formation ability of LSCs led by insulin-like growth factor 2 (IGF2) [121]. These findings suggested that IGF2/IGF1R/Nanog signaling cascade plays a critical role in regulating the LSCs proliferation while the overexpressed levels of Nanog trigger AML proliferation in mice and leukemogenesis in mice LSCs. This study supports that Nanog may be a possible candidate for leukemogenesis in rodents and might act like a Ventx ortholog.

These studies showed that Nanog and Ventx not only share functional similarities in maintaining cell pluripotency to delay cell-lineage commitments during embryonic development, but they are also similarly involved in cancer progression and leukemogenesis in humans.

## 5. Discussion and Conclusions

The Ventx and Vega families function downstream of BMP4/Smad1/5/8 signaling in vertebrates. BMP4/Smad1 upregulates the expression of *Ventx* and *Vega* families in the ventral regions of *Xenopus* and zebrafish embryos, respectively. The Ventx family is known for its transcriptional repressor activity, which drives the proper axial formation during embryonic development. The Ventx family not only inhibited dorsally expressed genes but also activated the transcription of ventral specific genes, leading to axial patterning and cell-fate determination in zebrafish and *Xenopus* [12,13,14,22,34,38,45,47,71,72,83,121]. Additionally, Ventx2.2 acted as a pluripotency regulator and maintained pluripotency in the VMZ. It could delay the blastula–gastrula transition during embryonic development of *Xenopus* [48,49]. Additionally, the inhibition of Ventx2.2 may trigger multiple cell-lineage commitments and gastrulation in *Xenopus* embryos.

In the Ventx family, the transactivation domain (TAD) of Ventx2.2 transactivated the Ventx genes, including *ventx1.1* and *ventx1.2*, in a LEF1/TCF-dependent manner in *Xenopus* [14,22,24,87]. The ectopic expression of Ventx2.2 inhibited β-cat levels, causing the induction of *gsk3β* mRNA, leading to embryonic ventralization in *Xenopus* [105]. However, a study reported that Ventx2.2 was phosphorylated and degraded in a GSK3β-independent manner during gastrulation in *Xenopus* [86]. Ventx2.2 might be involved in GSK3b-mediated cranial neural-crest-cell (CNC) migration rather than β-cat-dependent CNC migration in *Xenopus*. In humans, Ventx boosted cell proliferation and hematopoietic cell-terminal differentiation, including myeloid cell differentiation [18,32]. Studies have shown that Ventx also participated in monocyte–macrophage cell differentiation [50,51]. Ventx modulated TAM plasticity in TME to suppress the immune actions and macrophage differentiation against tumorigenesis [109]. An elevated level of retrovirally-engineered Ventx caused erythroleukemia and expanded the abnormal primitive erythroid cells in bone marrow progenitor cells, leading to leukemic progression in transplanted mice [32]. On the contrary, recent studies have revealed that Ventx may also act as an anti-cancer agent [54,115]. Ventx triggers apoptosis in p53^−/−^ knockout cell lines and activates cell senescence, which is followed by the activation of caspase-3, p53-p21, and p16^ink4^-Rb pathways. In p53^−/−^ cells, doxorubicin augmented the Ventx level to trigger apoptosis in a caspase-3-dependent manner. Therefore, the Ventx family acts as a key player in dorsoventral axial patterning, pluripotency, cell-fate determination, hematopoiesis, apoptosis, and CNC migration. Additionally, the inhibition of Ventx family members suppresses cell proliferation and tumor growth in humans.

## 6. Future Prospective for Targeting the GATA2- and Ventx-Associated Cancers

A study has shown that Ventx2 interacted with GATA2 to promote the functions of *ventx1.2* and *bmp4* in *Xenopus* [22]. Moreover, Ventx family members were highly expressed in VBIs during the development of *Xenopus* embryos. The expression of GATA2 was also directly controlled by BMP4/Smad1 signaling and remained high in VBIs to induce differentiation of the hematopoietic mesoderm in *Xenopus laevis* [41,122,123]. Elevated levels of *GATA2* were also reported in AML cell lines, patients’ samples, and tumors [124,125,126]. Cbfb-MYH11 is a chimeric product of core-binding-factor subunit-β (CBFB) and muscles myosin heavy chain 11 (MYH11). It was strongly associated with AML progression and triggered AML in humans. Elevated levels of GATA2 led to a poor prognosis and low survival in child AML, preleukemic Cbfb-MYH11 knock-in mice, and human inv(16) AML [124,125]. The RNAi- and K1717 (GATA2 inhibitor)-mediated inhibition of GATA2 notably diminished the expression of GATA2 targets—namely, Enah/Vasp-like AML markers, stem-like leukemia markers, and *WT1* (Wilms tumor1, implicated in AML)—in KG1a cells and in clinical patients’ AML cells, triggering apoptosis. The heterozygous knockout of GATA2^+/−^ in mice caused the inhibition of abnormal myeloid-progenitor-cell proliferation and differentiation and leukemia inhibition in Cbfb-MYH11 transgenic mice. Interestingly, transplanted leukemic cells drove leukemogenesis more aggressively in Cbfb-MYH11 and GATA2^+/−^ mice, but not in normal mice. GATA2 inhibition minimized the mutation frequency of genes associated with leukemogenesis and delayed the onset of leukemogenesis in Cbfb-MYH11 knock-in mice. Collectively, the knockdown of *GATA2* and *Ventx* attenuated cell proliferation and tumor growth, improved patient survival, and boosted the immunosuppression by drugs in tumor samples. Kumar et al. (2019) have reported that Ventx1.1 repressed the expression of *ventx2.2* and *ventx1.2* in a BMP4-independent manner in *Xenopus* embryos [80]. This inhibition was directly led by Ventx1.1 binding with the upstream region of *ventx2.2*, leading to *ventx2.2* inhibition in *Xenopus* embryos (unpublished data). A previous study has reported that overexpressing Ventx1.1 suppressed *GATA2* mRNA levels in the VMZ during hematopoiesis of *Xenopus* embryos [42]. A study demonstrated that Ventx1.1 interacted with Ventx2.2 during gastrulation of *Xenopus* embryos. However, the physiological outcome of this interaction remains unknown [127]. Since Ventx1.1 could interact with Ventx2.2 in *Xenopus*, it might be that the ectopic expression of Ventx1.1 inhibits the Ventx–GATA2 complex in humans and blocks GATA2- and Ventx2.1-induced leukemogenesis. Based on the collective evidence in *Xenopus* and humans, we concluded that Ventx1.1 may also inhibit the transcription of *Ventx* and *GATA2*, leading to the inhibition of human leukemogenesis. *Xenopus* Ventx1.1 could be a novel therapeutic pathway to target Ventx- and GATA2-associated cancers.

## Figures and Tables

**Table 1:** Summary of all HD proteins based on additional domains and conserved amino acid (AA) locations in DNA-binding homeobox domain. The sign (-) indicates the absence of glutamine residue at N + 1 position.

Superclass	Class	Subclass	AA Position in HD				AA in HD
	ANTP	HoxL	N = 5	N + 1	X = 50	X + 1	
			Arginine	-	Glutamine	Lysine	60
		NKL	Arginine	Glutamine	Glutamine	-	60
	PRD	Pax	Arginine	-	Serine	-	60
	PRD-like	PaxL	Arginine	-	Glutamine	(X + 3) Lysine	60
	LIM		Arginine	-	Glutamine	-	60
	ZF		Arginine	-	Glutamine	Arginine	60
	POU		Arginine	-	Cysteine	-	60
	HNF		Arginine	-	Alanine	-	60
	CUT	Cux	Arginine	-	Histidine	-	60
		ONECUT	Arginine	-	Methionine	-	60
		CMP	Arginine	-	Lysine		60
		SATB	Arginine	-	Glutamine	-	60
	PROS		Serine	-	Serine	-	60
	CERS		Serine	-	Arginine		60
	SIX/SO		Serine, threonine, valine	-	lysine	-	60
TALE	PBC		Arginine	-	Glycine	-	63
	MEIS		Arginine	-	Isoleucine	-	63
	TGIF		Arginine	-	Isoleucine	-	63
	IRO		Arginine	-	Alanine	-	63
	MKX		Lysine	-	Alanine	-	63

**Table 2:** Numbers of all HD proteins identified in humans, *Xenopus*, and zebrafish distributed across homeobox classes, subclasses, and families based on HD and additional domains.

Homeobox Class	Subclass	Number of Families of Different Classes			Number of Genes			References
		Human	*Xenopus*	Zebrafish	Human	*Xenopus*	Zebrafish	[68,69] http://homeodb.zoo.ox.ac.uk/ (accessed on 27 December 2021)
ANTP	HoxL	14	14	14	52	53	68	
	NKL	23	22	22	67	60	64	
PRD	Pax	3	3	3	7	5	9	
PRD-like	PaxL	28	18	18	91	32	44	
LIM		6	6	6	12	12	20	
POU		7	6	6	24	19	19	
HNF		2	2	2	3	3	6	
SINE		3	3	3	6	6	13	
TALE		6	6	6	30	16	29	
CUT		3	3	3	10	7	9	
PROS		1	1	1	2	2	3	
ZF		5	5	5	15	14	17	
CERS		1	1	1	5	2	3	
Others		3	3	1	9	19	18	
**Total**		105	93	91	333	250	322	

**Table 3:** The chromosomal localization and developmental roles of Ventx/Vega family members in *Xenopus*, zebrafish, and humans. * Some consider Ventx2.1 Xom/Xbr-1a, whereas others document Ventx2.2 as Xom/Xbr-1a. The genomic locations of a given gene were collected from the following genome browsers: *Xenopus* (http://www.xenbase.org) genome assembly version V.9.1 jbrowse, Zebrafish (http://www.zfin.org) genome assembly version GRCz11, and human (https://genome.ucsc.edu) genome assembly version GRCh38/hg38. NA indicates not known yet. All datasets are accessed on the 27 December 2021.

Names	Chromosomal Localization	Similarity with Human Ventx (%)	Developmental Roles	References
*Xenopus laevis*				
Venxt1.1/PV.1/Ventx1.1a/Ventx1.1b	Chr7L:21,842,291–21,845,156 Chr7S:16,920,567–16,923,476	59.02	Neural repressor, Spemann-organizer inhibitor, embryonic ventralization, hematopoiesis, axial patterning	[30,33,72]
Ventx1.2/Xvent1/Vent1/Vent-1/Xvent-1	Chr7L:21,802,386–21,807,247 Chr7S:16,883,662–16,887,492	44.95	Neural repressor, Spemann-organizer inhibitor, embryonic ventralization, axial patterning	[46,47,83]
Ventx2.1a/Ventx2.1b, Vox1, Vox, Xvent2	Chr7L:21,859,318–21,861,104 Chr7S:16,932,131–16,933,936	66.67	Neural repressor, Spemann-organizer inhibitor, embryonic ventralization, Ventx1.2 and ventx1.1 activator, hematopoiesis, axial patterning	[12,15,104]
Ventx2.2/Xom */Xvent-2/Xbr-1	Chr7L:21,830,246–21,832,823 Chr7S:16,908,607–16,911,052	66.67	Neural repressor, VMZ activator, induce GSK3β expression, axial patterning, pluripotency marker, neural crest migration	[27,28,87,90,105]
Ventx3.1/vent3	Chr7L-NA Chr7S:16,889,505–16,895,419	39.1	NA	[106,107]
Ventx3.2/Vex1	Chr7L:21,784,572–21,787,885 Chr7S:16,870,294–16,873,640	51.32	Neural repressor, VMZ activator, axial patterning	[37,102]
Zebrafish				
Vega1/Vox	Chr13:50,621,929–50,624,154	55	Neural repressor, VMZ, activator, axial patterning	[34]
Vega2/Vent1	Chr13:50,609,716–50,614,718	55	Neural repressor, VMZ activator, axial patterning	[45]
Ved	Chr10:44,997,062–45,002,196	49.12	Neural repressor, VMZ activator, axial patterning	[38]
Human				
Ventx	Chr10:133,237,855–133,241,928	100	Acute myeloid leukemia, cellular senescence, myeloid and macrophage differentiation, dendritic cell differentiation, immunosuppressor, apoptotic activator	[17,18]

## Data Availability

Not applicable.

## References

[1] Grainger R.M. (2012). *Xenopus* tropicalis as a model organism for genetics and genomics: Past, present, and future. Methods Mol. Biol..

[2] Blum M., Ott T. (2018). *Xenopus*: An Undervalued Model Organism to Study and Model Human Genetic Disease. Cells Tissues Organs.

[3] Jia Y., Liu X. (2020). Polyploidization and pseudogenization in allotetraploid frog *Xenopus laevis* promote the evolution of aquaporin family in higher vertebrates. BMC Genom..

[4] Hellsten U., Khokha M.K., Grammer T.C., Harland R.M., Richardson P., Rokhsar D.S. (2007). Accelerated gene evolution and subfunctionalization in the pseudotetraploid frog *Xenopus laevis*. BMC Biol..

[5] Elurbe D.M., Paranjpe S.S., Georgiou G., van Kruijsbergen I., Bogdanovic O., Gibeaux R., Heald R., Lister R., Huynen M.A., van Heeringen S.J. (2017). Regulatory remodeling in the allo-tetraploid frog *Xenopus laevis*. Genome Biol..

[6] Session A.M., Uno Y., Kwon T., Chapman J.A., Toyoda A., Takahashi S., Fukui A., Hikosaka A., Suzuki A., Kondo M. (2016). Genome evolution in the allotetraploid frog *Xenopus laevis*. Nature.

[7] Pollet N., Mazabraud A. (2006). Insights from *Xenopus* genomes. Genome Dyn..

[8] Gehring W.J., Affolter M., Burglin T. (1994). Homeodomain proteins. Annu. Rev. Biochem..

[9] Burglin T.R., Affolter M. (2016). Homeodomain proteins: An update. Chromosoma.

[10] Burglin T.R. (2011). Homeodomain subtypes and functional diversity. Subcell Biochem..

[11] Holland P.W., Booth H.A., Bruford E.A. (2007). Classification and nomenclature of all human homeobox genes. BMC Biol..

[12] Henningfeld K.A., Friedle H., Rastegar S., Knochel W. (2002). Autoregulation of *Xvent-2B*; direct interaction and functional cooperation of *Xvent-2* and *Smad1*. J. Biol. Chem..

[13] Kumar S., Umair Z., Yoon J., Lee U., Kim S.C., Park J.B., Lee J.Y., Kim J. (2018). Xbra and Smad-1 cooperate to activate the transcription of neural repressor *ventx1.1* in *Xenopus* embryos. Sci. Rep..

[14] Lee H.S., Lee S.Y., Lee H., Hwang Y.S., Cha S.W., Park S., Lee J.Y., Park J.B., Kim S., Park M.J. (2011). Direct response elements of BMP within the PV.1A promoter are essential for its transcriptional regulation during early *Xenopus* development. PLoS ONE.

[15] Lee H.S., Park M.J., Lee S.Y., Hwang Y.S., Lee H., Roh D.H., Kim J.I., Park J.B., Lee J.Y., Kung H.F. (2002). Transcriptional regulation of Xbr-1a/Xvent-2 homeobox gene: Analysis of its promoter region. Biochem. Biophys. Res. Commun..

[16] Faial T., Bernardo A.S., Mendjan S., Diamanti E., Ortmann D., Gentsch G.E., Mascetti V.L., Trotter M.W., Smith J.C., Pedersen R.A. (2015). Brachyury and SMAD signalling collaboratively orchestrate distinct mesoderm and endoderm gene regulatory networks in differentiating human embryonic stem cells. Development.

[17] Moretti P.A., Davidson A.J., Baker E., Lilley B., Zon L.I., D’Andrea R.J. (2001). Molecular cloning of a human Vent-like homeobox gene. Genomics.

[18] Rawat V.P., Arseni N., Ahmed F., Mulaw M.A., Thoene S., Heilmeier B., Sadlon T., D’Andrea R.J., Hiddemann W., Bohlander S.K. (2010). The vent-like homeobox gene VENTX promotes human myeloid differentiation and is highly expressed in acute myeloid leukemia. Proc. Natl. Acad. Sci. USA.

[19] Genthe J.R., Min J., Farmer D.M., Shelat A.A., Grenet J.A., Lin W., Finkelstein D., Vrijens K., Chen T., Guy R.K. (2017). Ventromorphins: A New Class of Small Molecule Activators of the Canonical BMP Signaling Pathway. ACS Chem. Biol..

[20] Little S.C., Mullins M.C. (2006). Extracellular modulation of BMP activity in patterning the dorsoventral axis. Birth Defects Res. C Embryo Today.

[21] Stickney H.L., Imai Y., Draper B., Moens C., Talbot W.S. (2007). Zebrafish *bmp4* functions during late gastrulation to specify ventroposterior cell fates. Dev. Biol..

[22] Friedle H., Knochel W. (2002). Cooperative interaction of *Xvent-2* and *GATA-2* in the activation of the ventral homeobox gene *Xvent-1B*. J. Biol. Chem..

[23] Messenger N.J., Kabitschke C., Andrews R., Grimmer D., Nunez Miguel R., Blundell T.L., Smith J.C., Wardle F.C. (2005). Functional specificity of the *Xenopus* T-domain protein Brachyury is conferred by its ability to interact with Smad1. Dev. Cell.

[24] Onichtchouk D., Gawantka V., Dosch R., Delius H., Hirschfeld K., Blumenstock C., Niehrs C. (1996). The *Xvent-2* homeobox gene is part of the BMP-4 signalling pathway controlling [correction of controling] dorsoventral patterning of *Xenopus* mesoderm. Development.

[25] Schuler-Metz A., Knochel S., Kaufmann E., Knochel W. (2000). The homeodomain transcription factor *Xvent-2* mediates autocatalytic regulation of BMP-4 expression in *Xenopus* embryos. J. Biol. Chem..

[26] Trindade M., Tada M., Smith J.C. (1999). DNA-binding specificity and embryological function of Xom (*Xvent-2*). Dev. Biol..

[27] Ladher R., Mohun T.J., Smith J.C., Snape A.M. (1996). Xom: A *Xenopus* homeobox gene that mediates the early effects of BMP-4. Development.

[28] Papalopulu N., Kintner C. (1996). A *Xenopus* gene, Xbr-1, defines a novel class of homeobox genes and is expressed in the dorsal ciliary margin of the eye. Dev. Biol..

[29] Zhong Y.F., Holland P.W. (2011). The dynamics of vertebrate homeobox gene evolution: Gain and loss of genes in mouse and human lineages. BMC Evol. Biol..

[30] Ault K.T., Dirksen M.L., Jamrich M. (1996). A novel homeobox gene PV.1 mediates induction of ventral mesoderm in *Xenopus* embryos. Proc. Natl. Acad. Sci. USA.

[31] Dale L., Wardle F.C. (1999). A gradient of BMP activity specifies dorsal-ventral fates in early *Xenopus* embryos. Semin. Cell Dev. Biol..

[32] Gentner E., Vegi N.M., Mulaw M.A., Mandal T., Bamezai S., Claus R., Tasdogan A., Quintanilla-Martinez L., Grunenberg A., Dohner K. (2016). VENTX induces expansion of primitive erythroid cells and contributes to the development of acute myeloid leukemia in mice. Oncotarget.

[33] Hwang Y.S., Seo J.J., Cha S.W., Lee H.S., Lee S.Y., Roh D.H., Kung Hf H.F., Kim J., Ja Park M. (2002). Antimorphic PV.1 causes secondary axis by inducing ectopic organizer. Biochem. Biophys. Res. Commun..

[34] Kawahara A., Wilm T., Solnica-Krezel L., Dawid I.B. (2000). Antagonistic role of vega1 and bozozok/dharma homeobox genes in organizer formation. Proc. Natl. Acad. Sci. USA.

[35] Lee S.Y., Lim S.K., Cha S.W., Yoon J., Lee S.H., Lee H.S., Park J.B., Lee J.Y., Kim S.C., Kim J. (2011). Inhibition of FGF signaling converts dorsal mesoderm to ventral mesoderm in early *Xenopus* embryos. Differentiation.

[36] Lee S.Y., Yoon J., Lee M.H., Jung S.K., Kim D.J., Bode A.M., Kim J., Dong Z. (2012). The role of heterodimeric AP-1 protein comprised of JunD and c-Fos proteins in hematopoiesis. J. Biol. Chem..

[37] Shapira E., Marom K., Yelin R., Levy A., Fainsod A. (1999). A role for the homeobox gene *Xvex-1* as part of the BMP-4 ventral signaling pathway. Mech. Dev..

[38] Shimizu T., Yamanaka Y., Nojima H., Yabe T., Hibi M., Hirano T. (2002). A novel repressor-type homeobox gene, ved, is involved in dharma/bozozok-mediated dorsal organizer formation in zebrafish. Mech. Dev..

[39] De Robertis E.M., Kuroda H. (2004). Dorsal-ventral patterning and neural induction in *Xenopus* embryos. Annu. Rev. Cell Dev. Biol..

[40] Hemmati-Brivanlou A., Thomsen G.H. (1995). Ventral mesodermal patterning in *Xenopus* embryos: Expression patterns and activities of BMP-2 and BMP-4. Dev. Genet..

[41] Maeno M., Mead P.E., Kelley C., Xu R.H., Kung H.F., Suzuki A., Ueno N., Zon L.I. (1996). The role of BMP-4 and GATA-2 in the induction and differentiation of hematopoietic mesoderm in *Xenopus laevis*. Blood.

[42] Xu R.H., Ault K.T., Kim J., Park M.J., Hwang Y.S., Peng Y., Sredni D., Kung H. (1999). Opposite effects of FGF and BMP-4 on embryonic blood formation: Roles of PV.1 and GATA-2. Dev. Biol..

[43] Friedle H., Rastegar S., Paul H., Kaufmann E., Knochel W. (1998). Xvent-1 mediates BMP-4-induced suppression of the dorsal-lip-specific early response gene *XFD-1*’ in *Xenopus* embryos. EMBO J..

[44] Imai Y., Gates M.A., Melby A.E., Kimelman D., Schier A.F., Talbot W.S. (2001). The homeobox genes vox and vent are redundant repressors of dorsal fates in zebrafish. Development.

[45] Kawahara A., Wilm T., Solnica-Krezel L., Dawid I.B. (2000). Functional interaction of vega2 and goosecoid homeobox genes in zebrafish. Genesis.

[46] Gawantka V., Delius H., Hirschfeld K., Blumenstock C., Niehrs C. (1995). Antagonizing the Spemann organizer: Role of the homeobox gene Xvent-1. EMBO J..

[47] Onichtchouk D., Glinka A., Niehrs C. (1998). Requirement for *Xvent-1* and *Xvent-2* gene function in dorsoventral patterning of *Xenopus* mesoderm. Development.

[48] Scerbo P., Girardot F., Vivien C., Markov G.V., Luxardi G., Demeneix B., Kodjabachian L., Coen L. (2012). Ventx factors function as Nanog-like guardians of developmental potential in *Xenopus*. PLoS ONE.

[49] Scerbo P., Marchal L., Kodjabachian L. (2017). Lineage commitment of embryonic cells involves MEK1-dependent clearance of pluripotency regulator *Ventx2*. eLife.

[50] Wu X., Gao H., Bleday R., Zhu Z. (2014). Homeobox transcription factor VentX regulates differentiation and maturation of human dendritic cells. J. Biol. Chem..

[51] Wu X., Gao H., Ke W., Giese R.W., Zhu Z. (2011). The homeobox transcription factor VentX controls human macrophage terminal differentiation and proinflammatory activation. J. Clin. Investig..

[52] Gartel A.L., Radhakrishnan S.K. (2005). Lost in transcription: p21 repression, mechanisms, and consequences. Cancer Res..

[53] Deng C., Zhang P., Harper J.W., Elledge S.J., Leder P. (1995). Mice lacking p21CIP1/WAF1 undergo normal development, but are defective in G1 checkpoint control. Cell.

[54] Wu X., Gao H., Ke W., Hager M., Xiao S., Freeman M.R., Zhu Z. (2011). VentX trans-activates *p53* and *p16ink4a* to regulate cellular senescence. J. Biol. Chem..

[55] Schmid M., Evans B.J., Bogart J.P. (2015). Polyploidy in Amphibia. Cytogenet. Genome Res..

[56] Becak M.L. (2014). Polyploidy and epigenetic events in the evolution of Anura. Genet. Mol. Res..

[57] Burgess S. (2016). Genomics: A matched set of frog sequences. Nature.

[58] Xie J., Nachabe A., Hathaway L.J., Farah B., Berbari B., Li Y., Brown T.C., Schmid J.L., Socola F., Saba N.S. (2021). The prognostic implications of tetraploidy/near-Tetraploidy in acute myeloid leukemia: A case series and systematic review of the literature. Leuk Lymphoma.

[59] Singh M.D., Jensen M., Lasser M., Huber E., Yusuff T., Pizzo L., Lifschutz B., Desai I., Kubina A., Yennawar S. (2020). NCBP2 modulates neurodevelopmental defects of the *3q29* deletion in Drosophila and *Xenopus laevis* models. PLoS Genet..

[60] Yaguchi K., Yamamoto T., Matsui R., Shimada M., Shibanuma A., Kamimura K., Koda T., Uehara R. (2018). Tetraploidy-associated centrosome overduplication in mouse early embryos. Commun. Integr. Biol..

[61] Tanaka K., Goto H., Nishimura Y., Kasahara K., Mizoguchi A., Inagaki M. (2018). Tetraploidy in cancer and its possible link to aging. Cancer Sci..

[62] Frade J.M., Lopez-Sanchez N. (2017). Neuronal tetraploidy in Alzheimer and aging. Aging.

[63] Tandon P., Conlon F., Furlow J.D., Horb M.E. (2017). Expanding the genetic toolkit in *Xenopus*: Approaches and opportunities for human disease modeling. Dev. Biol..

[64] Jonsdottir A.B., Stefansson O.A., Bjornsson J., Jonasson J.G., Ogmundsdottir H.M., Eyfjord J.E. (2012). Tetraploidy in *BRCA2* breast tumours. Eur. J. Cancer.

[65] Takeuchi M., Takeuchi K., Ozawa Y., Kohara A., Mizusawa H. (2009). Aneuploidy in immortalized human mesenchymal stem cells with non-random loss of chromosome 13 in culture. Vitr. Cell Dev. Biol. Anim..

[66] Nguyen H.G., Ravid K. (2006). Tetraploidy/aneuploidy and stem cells in cancer promotion: The role of chromosome passenger proteins. J. Cell Physiol..

[67] Burglin T.R. (1998). The PBC domain contains a MEINOX domain: Coevolution of Hox and TALE homeobox genes?. Dev. Genes. Evol..

[68] Yoon J., Kim J.H., Lee S.Y., Kim S., Park J.B., Lee J.Y., Kim J. (2014). PV.1 induced by FGF-Xbra functions as a repressor of neurogenesis in *Xenopus* embryos. BMB Rep..

[69] Zhong Y.F., Holland P.W. (2011). HomeoDB2: Functional expansion of a comparative homeobox gene database for evolutionary developmental biology. Evol. Dev..

[70] Zhong Y.F., Butts T., Holland P.W. (2008). HomeoDB: A database of homeobox gene diversity. Evol. Dev..

[71] Ault K.T., Xu R.H., Kung H.F., Jamrich M. (1997). The homeobox gene *PV.1* mediates specification of the prospective neural ectoderm in *Xenopus* embryos. Dev. Biol..

[72] Hwang Y.S., Lee H.S., Roh D.H., Cha S., Lee S.Y., Seo J.J., Kim J., Park M.J. (2003). Active repression of organizer genes by C-terminal domain of PV.1. Biochem. Biophys. Res. Commun..

[73] Kumar V., Park S., Lee U., Kim J. (2021). The Organizer and Its Signaling in Embryonic Development. J. Dev. Biol..

[74] Kumar V., Goutam R.S., Park S., Lee U., Kim J. (2021). Functional Roles of FGF Signaling in Early Development of Vertebrate Embryos. Cells.

[75] Kumar V., Umair Z., Kumar S., Lee U., Kim J. (2021). *Smad2* and *Smad3* differentially modulate chordin transcription via direct binding on the distal elements in gastrula *Xenopus* embryos. Biochem. Biophys. Res. Commun..

[76] Umair Z., Kumar S., Kim D.H., Rafiq K., Kumar V., Kim S., Park J.B., Lee J.Y., Lee U., Kim J. (2018). *Ventx1.1* as a Direct Repressor of Early Neural Gene *zic3* in *Xenopus laevis*. Mol. Cells.

[77] Yoon J., Kim J.H., Kim S.C., Park J.B., Lee J.Y., Kim J. (2014). PV.1 suppresses the expression of FoxD5b during neural induction in *Xenopus* embryos. Mol. Cells.

[78] Kumar V., Goutam R.S., Umair Z., Park S., Lee U., Kim J. (2021). Foxd4l1.1 Negatively Regulates Chordin Transcription in Neuroectoderm of *Xenopus* Gastrula. Cells.

[79] Kumar S., Umair Z., Kumar V., Kumar S., Lee U., Kim J. (2020). Foxd4l1.1 negatively regulates transcription of neural repressor ventx1.1 during neuroectoderm formation in *Xenopus* embryos. Sci. Rep..

[80] Kumar S., Umair Z., Kumar V., Lee U., Choi S.C., Kim J. (2019). Ventx1.1 competes with a transcriptional activator *Xcad2* to regulate negatively its own expression. BMB Rep..

[81] Pillemer G., Yelin R., Epstein M., Gont L., Frumkin Y., Yisraeli J.K., Steinbeisser H., Fainsod A. (1998). The *Xcad-2* gene can provide a ventral signal independent of BMP-4. Mech. Dev..

[82] Kumano G., Belluzzi L., Smith W.C. (1999). Spatial and temporal properties of ventral blood island induction in *Xenopus laevis*. Development.

[83] Sander V., Reversade B., De Robertis E.M. (2007). The opposing homeobox genes Goosecoid and Vent1/2 self-regulate *Xenopus* patterning. EMBO J..

[84] Rogers C.D., Archer T.C., Cunningham D.D., Grammer T.C., Casey E.M. (2008). Sox3 expression is maintained by FGF signaling and restricted to the neural plate by Vent proteins in the *Xenopus* embryo. Dev. Biol..

[85] Miyazaki A., Ishii K., Yamashita S., Nejigane S., Matsukawa S., Ito Y., Onuma Y., Asashima M., Michiue T. (2012). mNanog possesses dorsal mesoderm-inducing ability by modulating both BMP and Activin/nodal signaling in *Xenopus* ectodermal cells. PLoS ONE.

[86] Zhu Z., Kirschner M. (2002). Regulated proteolysis of Xom mediates dorsoventral pattern formation during early *Xenopus* development. Dev. Cell.

[87] Gao H., Wu B., Giese R., Zhu Z. (2007). Xom interacts with and stimulates transcriptional activity of LEF1/TCFs: Implications for ventral cell fate determination during vertebrate embryogenesis. Cell Res..

[88] Wroble B.N., Finkielstein C.V., Sible J.C. (2007). Wee1 kinase alters cyclin E/Cdk2 and promotes apoptosis during the early embryonic development of *Xenopus laevis*. BMC Dev. Biol..

[89] Trindade M., Messenger N., Papin C., Grimmer D., Fairclough L., Tada M., Smith J.C. (2003). Regulation of apoptosis in the *Xenopus* embryo by Bix3. Development.

[90] Wu B., Gao H., Le Y., Wu X., Zhu Z. (2018). Xom induces proteolysis of beta-catenin through GSK3beta-mediated pathway. FEBS Lett..

[91] Schuff M., Siegel D., Philipp M., Bundschu K., Heymann N., Donow C., Knochel W. (2012). Characterization of Danio rerio Nanog and functional comparison to *Xenopus* Vents. Stem. Cells Dev..

[92] Chambers I., Silva J., Colby D., Nichols J., Nijmeijer B., Robertson M., Vrana J., Jones K., Grotewold L., Smith A. (2007). Nanog safeguards pluripotency and mediates germline development. Nature.

[93] Scerbo P., Monsoro-Burq A.H. (2020). The vertebrate-specific *VENTX/NANOG* gene empowers neural crest with ectomesenchyme potential. Sci. Adv..

[94] Shakhova O., Sommer L. (2008). Neural crest-derived stem cells. StemBook.

[95] Fraser G.J., Hulsey C.D., Bloomquist R.F., Uyesugi K., Manley N.R., Streelman J.T. (2009). An ancient gene network is co-opted for teeth on old and new jaws. PLoS Biol..

[96] Miletich I., Yu W.Y., Zhang R., Yang K., Caixeta de Andrade S., Pereira S.F., Ohazama A., Mock O.B., Buchner G., Sealby J. (2011). Developmental stalling and organ-autonomous regulation of morphogenesis. Proc. Natl. Acad. Sci. USA.

[97] Nichols J.T., Pan L., Moens C.B., Kimmel C.B. (2013). barx1 represses joints and promotes cartilage in the craniofacial skeleton. Development.

[98] Sperber S.M., Dawid I.B. (2008). barx1 is necessary for ectomesenchyme proliferation and osteochondroprogenitor condensation in the zebrafish pharyngeal arches. Dev. Biol..

[99] Gao H., Le Y., Wu X., Silberstein L.E., Giese R.W., Zhu Z. (2010). VentX, a novel lymphoid-enhancing factor/T-cell factor-associated transcription repressor, is a putative tumor suppressor. Cancer Res..

[100] Kim B.M., Buchner G., Miletich I., Sharpe P.T., Shivdasani R.A. (2005). The stomach mesenchymal transcription factor Barx1 specifies gastric epithelial identity through inhibition of transient Wnt signaling. Dev. Cell.

[101] Kim B.M., Miletich I., Mao J., McMahon A.P., Sharpe P.A., Shivdasani R.A. (2007). Independent functions and mechanisms for homeobox gene *Barx1* in patterning mouse stomach and spleen. Development.

[102] Shapira E., Marom K., Levy V., Yelin R., Fainsod A. (2000). The *Xvex-1* antimorph reveals the temporal competence for organizer formation and an early role for ventral homeobox genes. Mech. Dev..

[103] Gilardelli C.N., Pozzoli O., Sordino P., Matassi G., Cotelli F. (2004). Functional and hierarchical interactions among zebrafish vox/vent homeobox genes. Dev. Dyn..

[104] Melby A.E., Clements W.K., Kimelman D. (1999). Regulation of dorsal gene expression in *Xenopus* by the ventralizing homeodomain gene Vox. Dev. Biol..

[105] Xie Y., Liu C. (2018). Xom, a ventralizing factor, regulates beta-catenin levels and cell fate. FEBS Lett..

[106] Leibovich A., Kot-Leibovich H., Ben-Zvi D., Fainsod A. (2018). ADMP controls the size of Spemann’s organizer through a network of self-regulating expansion-restriction signals. BMC Biol..

[107] Watanabe M., Yasuoka Y., Mawaribuchi S., Kuretani A., Ito M., Kondo M., Ochi H., Ogino H., Fukui A., Taira M. (2017). Conservatism and variability of gene expression profiles among homeologous transcription factors in *Xenopus laevis*. Dev. Biol..

[108] Trompouki E., Bowman T.V., Lawton L.N., Fan Z.P., Wu D.C., DiBiase A., Martin C.S., Cech J.N., Sessa A.K., Leblanc J.L. (2011). Lineage regulators direct BMP and Wnt pathways to cell-specific programs during differentiation and regeneration. Cell.

[109] Le Y., Gao H., Bleday R., Zhu Z. (2018). The homeobox protein VentX reverts immune suppression in the tumor microenvironment. Nat. Commun..

[110] Le Y., Gao H., Richards W., Zhao L., Bleday R., Clancy T., Zhu Z. (2020). VentX expression in tumor-associated macrophages promotes phagocytosis and immunity against pancreatic cancers. JCI Insight.

[111] Ino Y., Yamazaki-Itoh R., Shimada K., Iwasaki M., Kosuge T., Kanai Y., Hiraoka N. (2013). Immune cell infiltration as an indicator of the immune microenvironment of pancreatic cancer. Br. J. Cancer.

[112] Gao H., Wu X., Sun Y., Zhou S., Silberstein L.E., Zhu Z. (2012). Suppression of homeobox transcription factor VentX promotes expansion of human hematopoietic stem/multipotent progenitor cells. J. Biol. Chem..

[113] Elagib K.E., Goldfarb A.N. (2007). Oncogenic pathways of AML1-ETO in acute myeloid leukemia: Multifaceted manipulation of marrow maturation. Cancer Lett..

[114] Vargas A.C., Gray L.A., White C.L., Maclean F.M., Grimison P., Ardakani N.M., Bonar F., Algar E.M., Cheah A.L., Russell P. (2021). Genome wide methylation profiling of selected matched soft tissue sarcomas identifies methylation changes in metastatic and recurrent disease. Sci. Rep..

[115] Gao H., Wu B., Le Y., Zhu Z. (2016). Homeobox protein VentX induces p53-independent apoptosis in cancer cells. Oncotarget.

[116] Grubelnik G., Bostjancic E., Pavlic A., Kos M., Zidar N. (2020). NANOG expression in human development and cancerogenesis. Exp Biol. Med..

[117] Nagel S., Scherr M., MacLeod R.A.F., Pommerenke C., Koeppel M., Meyer C., Kaufmann M., Dallmann I., Drexler H.G. (2019). NKL homeobox gene activities in normal and malignant myeloid cells. PLoS ONE.

[118] Brandner S. (2010). Nanog, Gli, and p53: A new network of stemness in development and cancer. EMBO J..

[119] Zbinden M., Duquet A., Lorente-Trigos A., Ngwabyt S.N., Borges I., Ruiz i Altaba A. (2010). NANOG regulates glioma stem cells and is essential in vivo acting in a cross-functional network with GLI1 and p53. EMBO J..

[120] Wang S., Fan H., Xu J., Zhao E. (2018). Prognostic implication of NOTCH1 in early stage oral squamous cell cancer with occult metastases. Clin. Oral Investig..

[121] Schier A.F. (2001). Axis formation and patterning in zebrafish. Curr. Opin. Genet. Dev..

[122] Mimoto M.S., Kwon S., Green Y.S., Goldman D., Christian J.L. (2015). GATA2 regulates Wnt signaling to promote primitive red blood cell fate. Dev. Biol..

[123] Kelley C., Yee K., Harland R., Zon L.I. (1994). Ventral expression of GATA-1 and GATA-2 in the *Xenopus* embryo defines induction of hematopoietic mesoderm. Dev. Biol..

[124] Yang L., Sun H., Cao Y., Xuan B., Fan Y., Sheng H., Zhuang W. (2017). GATA2 Inhibition Sensitizes Acute Myeloid Leukemia Cells to Chemotherapy. PLoS ONE.

[125] Saida S., Zhen T., Kim E., Yu K., Lopez G., McReynolds L.J., Liu P.P. (2020). Gata2 deficiency delays leukemogenesis while contributing to aggressive leukemia phenotype in Cbfb-MYH11 knockin mice. Leukemia.

[126] Luesink M., Hollink I.H., van der Velden V.H., Knops R.H., Boezeman J.B., de Haas V., Trka J., Baruchel A., Reinhardt D., van der Reijden B.A. (2012). High GATA2 expression is a poor prognostic marker in pediatric acute myeloid leukemia. Blood.

[127] Hwang Y.S., Chae J.P., Kim D.S., Park K.M., Bae Y.C., Park M.J. (2007). Screening of Interacting Proteins with PV. 1 as Downstream Factors of BMP Signal. Korean J. Anat..

